# Mapping Critical Residues in ATG11’s Coiled-Coil 2 Domain that Block Multiple Interactions and Disrupt Selective Autophagy

**DOI:** 10.3389/fcell.2021.775364

**Published:** 2022-01-18

**Authors:** Mitchell D. Meyer, Jasmine Winzeler, Sophia M. Taylor, Alex Kilgore, Kimberly Edicha, Chase Chitwood, Zachary Spearin, S. K. Nadia Rahman Silvia, Ronith Chakraborty, Jesse E. Smith, Bridget Kennedy, Carson Zois, Hayley Cawthon, Mukiri Gilruth, Steven K. Backues

**Affiliations:** Department of Chemistry, Eastern Michigan University, Ypsilanti, MI, United States

**Keywords:** Atg1, Atg9, Atg11, Atg19, yeast-2-hybrid, coimmunoprecipitation, directed mutagenesis, selective autophagy

## Abstract

Selective autophagy is a conserved subcellular process that maintains the health of eukaryotic cells by targeting damaged or toxic cytoplasmic components to the vacuole/lysosome for degradation. A key player in the initiation of selective autophagy in *S. Cerevisiae* (baker’s yeast) is a large adapter protein called Atg11. Atg11 has multiple predicted coiled-coil domains and intrinsically disordered regions, is known to dimerize, and binds and organizes other essential components of the autophagosome formation machinery, including Atg1 and Atg9. We performed systematic directed mutagenesis on the coiled-coil 2 domain of Atg11 in order to map which residues were required for its structure and function. Using yeast-2-hybrid and coimmunoprecipitation, we found only three residues to be critical: I562, Y565, and I569. Mutation of any of these, but especially Y565, could interfere with Atg11 dimerization and block its interaction with Atg1 and Atg9, thereby inactivating selective autophagy.

## Introduction

Selective macroautophagy (or, commonly, selective autophagy) is an evolutionarily conserved pathway that maintains cellular homeostasis. The pathway functions by sequestering cargo from the cytoplasm into the lysosome (in animals) or vacuole (in other eukaryotes). Most cargo are materials harmful to the cell in need of degradation. Autophagy pathways are named after the contents of their cargo. Mitophagy, for example, disposes of damaged mitochondria, xenophagy targets intracellular pathogens, and aggrephagy degrades large protein aggregates (Gatica et al., 2018). Selective autophagy plays a pivotal role in cellular health by clearing damaged proteins and organelles such as those that cause neurodegenerative diseases like Parkinson’s and Alzheimer’s (Scrivo et al., 2018; Wu et al., 2018). Understanding the mechanistic detail of selective autophagy through determining functions of autophagy-related proteins will hopefully lead to the development of impactful therapeutic agents with benefit to human health. The primary model used for studying the mechanistic details of autophagy is *Saccharomyces cerevisiae,* baker’s yeast. The adapter protein Atg11 is a key player in selective autophagy in yeast.

Selective autophagy initiation is characterized by envelopment of the cargo materials in a double membraned vesicle termed the autophagosome, which is built *de novo* around the cargo in response to the presence of specific receptor proteins (Zaffagnini and Martens, 2016; Gatica et al., 2018). These receptor proteins recognize the cargo with one end and with their distal end bind the autophagic proteins Atg8 and Atg11. Atg8 lines the membrane of the forming autophagosome, while Atg11 plays a central role in selective autophagosome initiation. Atg11 has homology to the human proteins RB1CC1/FIP200 and HTT/Huntingtin, both of which may play similar roles in human autophagy (Li et al., 2014; Ochaba et al., 2014; Turco et al., 2019).

Atg11 is an important component of the Autophagy Initiation Complex (AIC), also known as the Atg1 kinase complex. This critical protein complex starts the process of building the autophagosome by recruiting vesicles carrying the transmembrane protein Atg9 (He et al., 2006; Chang and Huang, 2007; Sekito et al., 2009; Suzuki et al., 2015). The fusion of these first vesicles is thought to be the genesis of the autophagosomal double membrane (Rao et al., 2016; Hurley and Young, 2017). Atg1 is a protein kinase which, along with its obligate partner Atg13, plays both a structural and a regulatory role in autophagosome formation (Abeliovich et al., 2000; Kamada et al., 2000; Cheong et al., 2008). Atg11 activates Atg1 by clustering it together on the surface of the cargo (Kamber et al., 2015; Torggler et al., 2016) and also directly recruits Atg9 to the site of autophagosome formation (He et al., 2006; Yao et al., 2020).

The process of autophagosome initiation is currently best understood during nitrogen-starvation-induced non-selective autophagy. Under these conditions the AIC is organized by Atg17, a large coiled-coil protein that has been crystalized along with its regulatory subunits (Atg29 and Atg31) and found to form a large S-shaped dimer (Ragusa et al., 2012). These dimers are then thought to be linked by Atg1 and Atg13 into larger oligomers providing the scaffold for the fusion of the first Atg9-containing vesicles (Köfinger et al., 2015; Hurley and Young, 2017). However, Atg17 is not required for selective autophagy. Instead, Atg11 is thought to play the key organizational role during selective autophagy (Kamada et al., 2000). In fact, Atg11 and Atg17 compete for binding with Atg9, contributing to the transition of autophagosome formation from selective (cargo-driven) to nonselective during starvation (Matscheko et al., 2019). Unfortunately, little structural data is available on Atg11, leaving critical gaps in our understanding of selective autophagy initiation.

Atg11 has been shown to form a parallel homodimer, likely held together by coiled-coil interactions (Suzuki and Noda, 2018; Margolis et al., 2020). The binding sites of Atg11’s many partner proteins have been roughly mapped via yeast-2-hybrid (Y2H) and coimmunoprecipitation (CoIP) assays using constructs containing deletions of large regions of Atg11. In particular, the C-Terminal Region (CTR) of Atg11 is the location of its interaction with the cargo receptors Atg19 and Atg32 (Yorimitsu and Klionsky, 2005; Aoki et al., 2011). The Atg11 CTR is loosely conserved in human FIP200, which uses this “claw” domain to bind to human cargo receptors such as p62 and NDP52 (Ravenhill et al., 2019; Turco et al., 2019). FIP200, like Atg11, is a large parallel dimer and contains a coiled-coil domain in its middle section that also binds NPD52 as well as interacting with negatively charged lipid membranes (Shi et al., 2020a). The N-terminus of FIP200 is intrinsically disordered until the binding of Atg13, at which time it folds into a crescent-shaped dimer which binds to ULK1, the mammalian homology of Atg1 (Shi et al., 2020b). Despite these functional similarities, there is no significant sequence homology between Atg11 and FIP200 outside of the CTR. The middle region of Atg11; however, does include two predicted coiled-coil (CC) regions named CC2 and CC3 (Yorimitsu and Klionsky, 2005). These are required for the binding of Atg11’s other partners, as well as Atg11’s self-interaction.

A recent crystal structure of the Atg11 CC3 region (a.a. 699–800) has shown that it forms a parallel dimeric coiled-coil. The central 30 a.a. of CC3 are well-ordered, held in place by the characteristic hydrophobic interactions provided by the heptad repeats. However, the outer portions of CC3 are poorly ordered, suggesting significant flexibility. In contrast to the lengthy CC3, CC2 is only 40 a.a. in length and is more weakly predicted to form a coiled-coil. Atg1, Atg9, Atg20, Atg29, and Ypt1 – but, critically, not Atg19 – have all been reported to require CC2 for their interaction, suggesting that CC2 is a key binding site (Yorimitsu and Klionsky, 2005; He et al., 2006; Lipatova et al., 2012). Atg1 binding also requires CC3, while Atg9 binding requires the entire N-terminus (Yorimitsu and Klionsky, 2005; He et al., 2006). In addition, Atg11 self-interaction has been reported to require CC2 (Yorimitsu and Klionsky, 2005) while there is conflicting data on whether or not it requires CC3 (Yorimitsu and Klionsky, 2005; Margolis et al., 2020).

Overall, while the CC2 region is a critical component of selective autophagy, it has previously eluded mechanistic characterization. Elucidation of Atg11’s structure and interactions will be crucial to understanding the role Atg11 plays in organizing the initiation of selective autophagy. Therefore, we identified the CC2 region as our target for further investigation by mutational analysis.

## Methods

All media components were purchased from ForMedium (Norfolk, United Kingdom); 3-amino-1,2,4-triazole (3AT) and G418 Sulfate were purchased from P212121; all other chemicals were from P212121 or VWR. Final figures were assembled using Adobe Photoshop and Illustrator.

### Plasmid Construction

Plasmids and their sources are listed in Supplementary Table S1. Primers were purchased from Integrated DNA Technologies; primer sequences are available upon request. PCR was performed using Phusion DNA Polymerase (NEB) and the products were purified using the NucleoSpin DNA and PCR Clean up Kit (Macherey-Nagel). DpnI (NEB) treatment was used as needed prior to clean up to reduce template background. All plasmids were verified by sequencing prior to use.

pGAD-ATG11, pGAD-ATG11CTR, and pGAD-ATG11∆CTR were prepared by Ligation Independent Cloning (LIC) (Aslanidis and de Jong, 1990). pGAD-C1 and the ATG11 coding sequence (full length, a.a. 854-1178 or a.a. 1-858, respectively) were amplified with primers containing LIC sites and treated with T4 polymerase. The plasmids were annealed and transformed into *E. coli*. The resulting construct contained ATG11 in frame with the Gal4-AD sequence.

The pGAD-ATG11 e/g, b/c/f, and a/d “Full” mutants were generated by InFusion cloning. PCR with primers flanking the CC2 region was used to amplify the entire pGAD-ATG11 vector without the CC2 region. Linear inserts (“gblocks”) comprising CC2 with the desired mutations plus 15 bp of homology on either side were purchased from IDT. The amplified vector and the insert fragment were joined and re-circularized using the InFusion EcoDry cloning system (Takara Bio).

The pGAD-ATG11 a/d-2, -3, and -4 double mutants were generated using inverse PCR with flanking phosphorylated primers. The primers were designed to anneal to the template plasmid tail-to-tail and run in reverse directions, with one primer containing the mutations of interest. The resulting linear PCR fragment was circularized via ligation with T4 ligase (NEB).

pGAD-ATG11 I569E was custom generated by Genscript. All other pGAD-ATG11 single mutants were generated by site-directed mutagenesis using inverse PCR and InFusion cloning (Takara Bio) (Takara Bio, 2021). Complementary mutagenic primers were designed with 15-20 bp of overlap at the site of the desired mutations. The resulting linear PCR fragment was circularized via an InFusion reaction.

To generate pGBDU-ATG11 mutant vectors, ATG11 containing the desired mutation or deletion was amplified out of the respective pGAD-ATG11 vectors with primers containing LIC sites. The empty pGBDU-C3 plasmid was likewise amplified with primers with LIC sites, and the two were joined by LIC.

To generate the pRS416-Cu-GFP-ATG11 mutants, pRS416-Cu-GFP-ATG11 was digested with NruI, which cuts twice in ATG11: one cut upstream of CC2 and one cut downstream. To obtain the mutated insert, the target CC2 region was amplified from the respective pGAD-ATG11 mutant plasmid using primers with 15 bp of homology on either side of the NruI cut sites. The NruI-digested vector and the CC2 mutant insert were joined via an InFusion reaction.

pMCSG10-ATG11CC2-3 and its mutants were generated by LIC. pMCSG10 was amplified with primers annealing just past the GST-TEV sequence to generate linear pMCSG10 with LIC-compatible ends. The sequence coding for the CC2-3 region (a.a. 537-853) was amplified with primers that added LIC-compatible ends from genomic DNA (for WT ATG11) or from the respective pGAD-ATG11 mutant plasmid. These fragments were then cloned into pMCSG10 in-frame with the GST-TEV sequence to create the final product.

pRS414-Cu-ATG9-PA was generated by InFusion cloning from three fragments. The first fragment was pRS414 linearized via a double digest with BamHI and EcoRI. The second fragment was the *Cu* promoter (291 bp of sequence immediately 5′ of the *CUP1* coding sequence), amplified from genomic DNA; the 5′ primer added 15 bp of homology to the linearized vector (BamHI side). Fragment 3 was ATG9-PA with a TEF terminator, amplified from pRS314-ATG9-PA-TEF (a generous gift of Dr. Klionsky, University of Michigan) with a 5′ primer adding 15 bp of homology to the Cu promoter and a 3’ primer adding 15 bp of homology to the linearized vector (EcoRI side). All three fragments were joined into a circular vector via InFusion cloning.

### Strain Construction

The strains used in this study are listed in Supplementary Table S2. The *atg11Δ* and ATG1-PA strains were generated using the methods of Longtine et al. (1998) and verified by PCR with primers flanking the ATG11 or ATG1 locus, respectively.

### Yeast Culture Conditions

Yeast were grown in SMD (0.69 g/L yeast nitrogen base w/o a.a., 1x amino acids and nucleotides, 2% w/v glucose) with the appropriate nutritional selection to maintain plasmids (-ura, -trp, and/or -leu) at 30°C with shaking at 300 RPM.

### Yeast-2-Hybrid

The WT Y2H strain PJ69-4A or the Multiple-Knockout (MKO) Y2H strain YCY149 was transformed sequentially with a BD vector containing an Atg11 binding partner and an AD vector containing wild type or mutant Atg11. (For the Atg11-Atg19 screen, Atg11 and its mutants were instead in the BD vector and Atg19 in the AD vector due to autoactivation seen with BD-Atg19). Yeast containing both plasmids were patched sequentially onto the appropriate selective (see Supplementary Table S3) and nonselective (SMD -ura -leu) plates. Two independently generated plasmids of each Atg11 mutant were used for each screen to eliminate false positives, and each screen was performed at least twice. Plates were imaged after 3–5 days of growth using a BioRad Chemidoc XRS + system. Quantitative log-phase liquid culture Y2H assays were performed as described by M. Montanto (Montano, 2001).

### TCA Precipitation

1 OD of yeast were harvested by centrifugation (5 min, 2000 g), resuspended in 10% trichloroacetic acid (TCA), incubated 30 min on ice, and pelleted 5 min, 16,000 g at 4°C. The resulting TCA-precipitated protein pellets were washed once with ice-cold acetone, air dried, and stored at −20°C for analysis. Prior to Western Blotting they were resuspended in 1xSSB supplemented with 0.25 M Tris pH 6.8.

### Coimmunoprecipitation

To generate spheroplasts, cultures were grown to log phase in SMD -ura, -trp, harvested by centrifugation, resuspended in 10 m of softener buffer (100 mM Tris-HCl pH 9.5, 10 mM DTT) and shaken 10 min (180 RPM, RT). Cells were harvested and resuspended in 5 mls of spheroplasting medium (1X YNB, 2% w/v glucose, 0.5% w/v casamino acids, 1X -trp -ura amino acid stock, 1.2 M sorbitol, 20 mM Tris-Cl pH 7.5) and treated with 40 μg/OD of Zymolyase 20T (US Biologicals) with gentle shaking at (180 RPM, 30°C) until spheroplasting was >80% complete (15–30 min). The spheroplasts were washed by centrifugation (800xg, 10 min) through 6 ml of 1.8 M sorbitol.

Spheroplasts were resuspended to ∼50 OD/ml in lysis buffer (20 mM Hepes-KOH pH 6.8, 150 mM KOAc, 5 mM MgOAc, 250 mM sorbitol, 0.5% TX-100 with 1 mM PMSF and 1x ProBlock Gold Yeast/Fungi protease inhibitor cocktail (Goldbio) added just before use) and passed through a 13 mm filter with 3.0 μm pore size (Whatman) using a 5 ml syringe and a 13 mm filter holder apparatus (Sartorius). The lysate was cleared twice at 500 g for 5 min.

For PA-tag pulldowns, 1 ml of cleared lysate was incubated at 4°C for 1 h with gentle rotation with 40 μl of 50% slurry of IgG-Sepharose 6 Fast Flow (GE Healthcare). The resin was pelleted, washed 3 to 5 times with lysis buffer (without PMSF or protease inhibitor cocktail), and once with lysis buffer without TX-100, then resuspended in 50 ul 2xSSB to generate the pulldown fraction. For HA-tag pulldowns, 1 ml of cleared lysate was incubated at 4°C for 2 h with gentle rotation with 1 µl of anti-HA antibody (rabbit polyclonal ab9110, Abcam). 40 µl of 50% slurry of PA-Sepharose was added to the lysate and incubated for 1 h at 4°C with gentle rotation. The resin was pelleted, washed 5 times with lysis buffer, and once with lysis buffer without TX-100, and resuspended in 50 µl 2xSSB to generate the pulldown fraction. Samples of the lysate and pulldowns were analyzed by western blotting.

### Western Blotting

Western blotting was performed using a Bio-Rad SDS-PAGE and tank transfer system according to standard procedure. Samples in SSB were heated for 5 min at 95°C and pelleted at 13,000 g at room temperature prior to the loading. For the transfer, PVDF membrane (Millipore) and Towbin-EtOH (15 mM Tris, 192 mM glycine, 9.5% v/v ethanol) was used. Blocking and antibody incubations were performed in 4% w/v nonfat dry milk in TBS w/0.1% v/v Tween. Blots were detected with Luminata Crescendo Western HRP substrate (Millipore) and imaged on a BioRad Chemidoc XRS+ system.

For measurement of the stability of the Atg11 Y2H mutants, YCY149 containing the appropriate Atg11 mutants in pGAD or pGBDU was grown to saturation in SMD -ura or SMD -ura, -leu, as appropriate. The antibodies used were: Mouse anti-GAL4 AD (14-7E10G10; Abcam), 1:1,000; Mouse anti-GAL4 BD (15-6E10A7; Abcam), 1:500; Mouse anti-Pgk1 (22C5D8; Abcam) 1:10,000; Secondary peroxidase-conjugated goat anti-mouse (Jackson ImmunoResearch 115-035-003) 1:5,000.

For measurement of Ape1 processing and the stability of the GFP-Atg11 mutants, SEY6210 atg11Δ containing pRS416-Cu-GFP-ATG11 with or without various mutations was grown to log phase in SMD -ura and starved for 3 h in SD-N (1x YNB w/o (NH_3_)_2_SO_4_, 2% glucose). Additional antibodies used were rabbit polyclonal anti-Ape1 (a generous gift of Dr. Klionsky, University of Michigan), 1:5,000; and mouse anti-GFP (JL-8; Takara Bio), 1:500.

The Atg1-PA and Atg9-PA pulldown blots were also probed with 1:500 mouse anti-GFP (JL-8) and 1:5000 secondary peroxidase-conjugated goat anti-mouse, thus recognizing both the GFP and the PA tag. The Atg19-PA blots were cut at the 72 kDa ladder and the top half was probed with anti-GFP followed by secondary while the bottom half was probed with 1:25,000 rabbit peroxidase-anti-peroxidase (Jackson ImmunoResearch, cat. # 323-005-024). The HA-tag pulldown blots were probed with 1:5000 anti-HA antibody (rabbit polyclonal ab9110, Abcam) and 1:5000 secondary peroxidase-conjugated goat anti-rabbit antibody (Jackson ImmunoResearch, cat. # 111-035-003)

Quantification of all western blots was performed on the original .scn files with ImageJ (v1.51S, FIJI bundle) (Schindelin et al., 2012; Schneider et al., 2012) using only bands in the linear range (visible but non-saturated). For quantification of CoIP samples, the GFP band in the negative control lane(s) (no GFP-Atg11 and/or no bait, as appropriate) were subtracted from the GFP bands in the CoIP lanes to remove the effect of nonspecific binding of GFP-Atg11 or background luminescence from the detection of the bait. The GFP band was then divided by the band for the bait (HA or PA, as appropriate), and then each lane was divided by the WT lane in order to normalize all samples to WT. This was done independently for each replicate, and then the results of all replicates (≥3) were combined. Quantification of the lysates was similar except that no negative control values were subtracted. Graphs were generated using R Studio (RStudio Team, 2020). All graphs show mean ± the 95% confidence interval; the significance of each mutant was measured against the normalized WT value of 100% using a one-sample *t*-test.

### Atg11 CC2-3 Heterologous Expression and Stability Testing

GST-Atg11CC2-3 (a.a. 537-853) with various CC2 mutations was produced in *E. coli.* BL21 cells containing pMCSG10-ATG11CC2-3 were autoinduced (Studier, 2005) for 30 h with shaking at 21°C, 300 RPM. Three ml of culture was harvested (5 min 10,000 g) and washed in 1 ml of buffer A (50mM HEPES pH8, 500 mM NaCl, 10% v/v glycerol, 5 mM EDTA). The washed pellet was then resuspended to 16 OD/ml in Lysis Buffer (Buffer A with 10mM 2-mercaptoethanol, 1 mM PMSF and 1X Goldbio GB-330-5 protease inhibitor cocktail). Cells were disrupted by sonication (five 12 s pulses, power level 5 on a Virsonic 100 probe sonicator (Virtis)), and TX-100 was added to a final concentration of 1%. 100 ul of lysate was collected and 100 ul of 2X SSB was added for the total fraction. The remainder of the lysate was centrifuged for 10 min, 16,000 g at 4°C, 100 ul of supernatant was collected and 100 ul of 2X SSB was added for the soluble fraction. Samples were analyzed by SDS-PAGE and Coomassie staining.

### Sequence Alignment

Protein sequences for Atg11 homologues from 23 species of budding yeast, order Saccharomycetales, were downloaded from the NCBI Genome Data Viewer (NCBI Resource Coordinators, 2018). Species were chosen that represented the diversity of the order, with 12 (including *S. Cerevisiae*) from family Saccharomycetacea and 11 from other families within Saccharomycetales, including multiple representatives of both the CTG clade and the methyltrophs (Dujon and Louis, 2017). If the Atg11 homologue in a given species had not been annotated, it was identified by protein BLAST (Altschul et al., 1990) of *S. Cerevisiae* Atg11 against the genome of that species. First, the full sequence of all 23 proteins were aligned using Clustal Omega (Goujon et al., 2010; Sievers et al., 2011) with default parameters. This alignment was used to select the 35 amino acids of each homologue that corresponded to CC2, and then just those 35 a.a. were used to perform a second alignment in Clustal Omega which was visualized using Jalview (Waterhouse et al., 2009).

### Comparison to Predicted Structures

The AlphaFold-predicted structural model for Q12527 (*Sc*Atg11) was viewed in the AlphaFold Protein Structure Database at https://alphafold.ebi.ac.uk/entry/Q12527, which showed the confidence of the predictions (Jumper et al., 2021). The model, AF-Q12527-F1-model_v1.pdb, was downloaded, viewed, and aligned with the crystal structure of Atg11CC3 (PDB 6VZF) (Margolis et al., 2020) using PyMol v2.1.1.

## Results

### Isoleucine 562 and Tyrosine 565 Are Critical for the Interaction of Atg11 With Atg1

**FIGURE 1:**
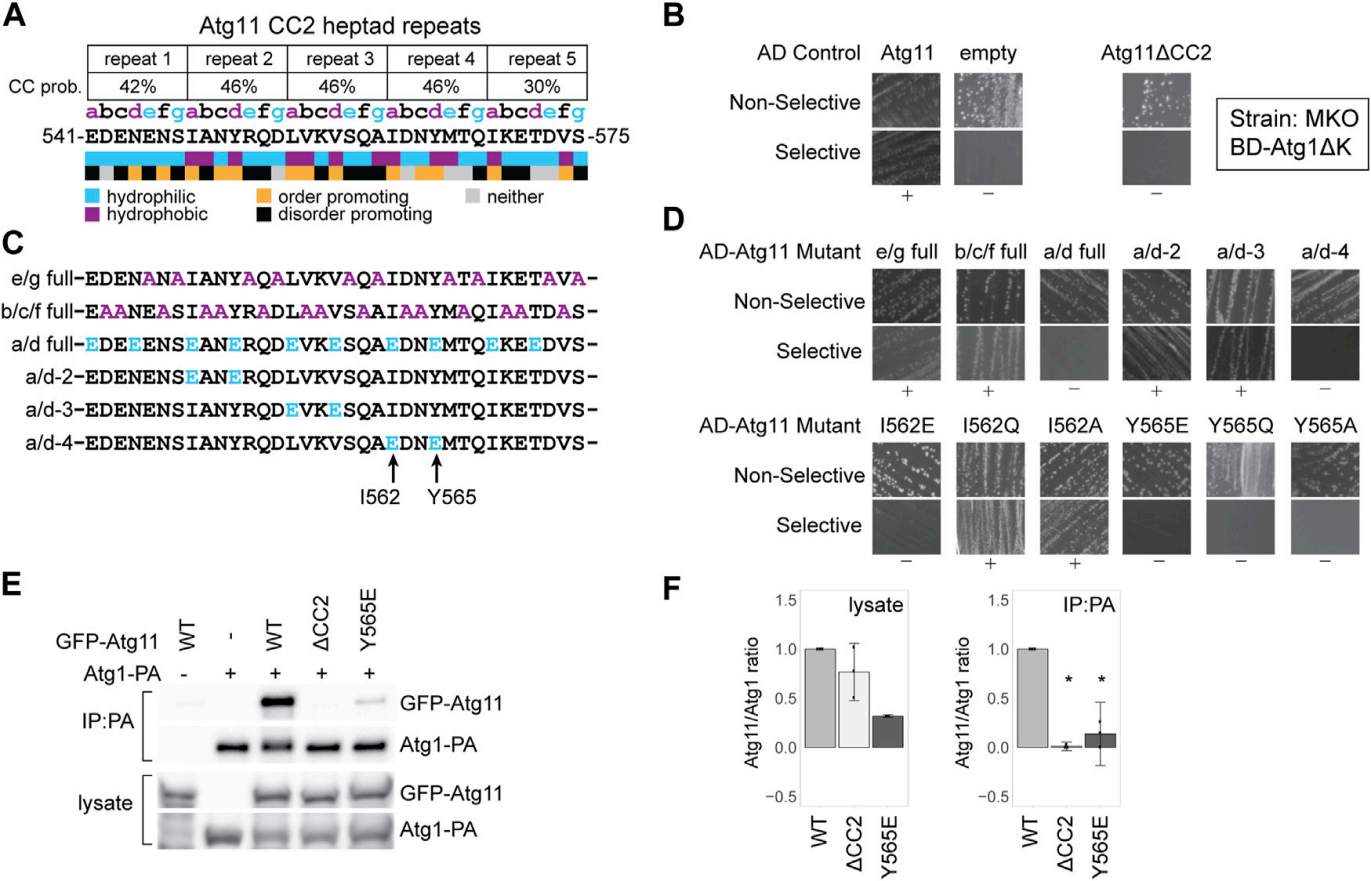
Atg11’s interaction with Atg1 is dependent on tyrosine 565 in its CC2 region. **(A)** Schematic of the five heptad repeats that form the core of the Atg11 CC22 domain. The Paircoil2-predicted probability of each repeat forming a coiled-coil is shown, and each residue is assigned a register (a, b, c, d, e, f, or g) based on those predictions. Colored letters indicate the expected chemical nature of a residue in that position of a heptad repeat. Colored boxes indicate the actual chemical nature of each residue and its propensity to promote or disorder. **(B)** Y2H results of BD-Atg1ΔK and AD-Atg11 in the multiple knockout (MKO) strain. Atg11ΔCC2 is Atg11Δ^536–576^; all others are full length Atg11. Non-selective media is SMD -ura, -leu, while selective media is SMD -ura, -leu, -ade. “+” indicates growth and “−” indicates no growth on selective media in multiple replicates. **(C)** Schematics of the CC2 region of the indicated Atg11 mutants. **(D)** Yeast-2-hybrid results of BD-Atg1ΔK with the indicated AD-Atg11 mutants. Assay performed as in (b). **(E)** CoIP assays showing the ability of endogenously driven Atg1-PA to precipitate overexpressed GFP-Atg11 and its mutants; **(F)** Quantification of CoIP results from **(E)**: four independent replicates, with the background from the negative control subtracted, each normalized to its respective WT sample. Errors bars are 95% CI; * = *p* <0.05 vs WT. Full, uncropped Y2H plates and CoIP blots are available in Supplementary Figure S5A and Supplementary Figure S7A, respectively.

Sequences forming coiled-coils can be recognized by their characteristic “heptad repeat”, an ordered pattern of hydrophobic and hydrophilic residues. Atg11’s CC2 region (residues 536–576) generally follows this pattern, although deviations from it cause the coiled-coil prediction (via the Paircoil2 algorithm (McDonnell et al., 2006)) to only be of moderate confidence (Figure 1A). The presence of many disorder-promoting residues in this region suggest that it may be loosely folded or undergo a disordered-to-ordered transition, similar to the outer regions of CC3. Indeed, CC2, along with other regions of Atg11, scores high on a combined metric of predicted intrinsic disorder (Popelka et al., 2014).

To better understand the contribution of CC2 to Atg11’s recruitment of the AIC, we tested the ability of Atg11 CC2 mutants to interact with Atg1 using a Y2H assay. BD-Atg1∆K, which lacks the C-terminal kinase domain, was used instead of full length Atg1 due to reported autoactivation by full length BD-Atg1 (Yorimitsu and Klionsky, 2005). The assay was performed in a multiple knockout (MKO) background, where 25 *ATG* genes have been deleted, in order to test for dependence on any other components of the autophagic machinery (Cao et al., 2009). BD-Atg1∆K shows a robust interaction with full length, wild type AD-Atg11 in this strain suggesting that the Atg1-Atg11 interaction is direct, or at least independent of any other ATG proteins. This interaction was absent when AD-Atg11∆CC2 was used, verifying the essential contribution of the CC2 region (Figure 1B).

We then systematically mutagenized CC2, beginning with mutants targeting specific positions of the heptad repeat pattern along the entire 40 a.a. length of the CC2. The “e/g full” mutant had alanines substituted at every hydrophilic “e” or “g” position; the “b/c/f full” mutant had alanines substituted at every “b”, “c”, or “f” position; and the “a/d full” mutant had glutamates substituted for every hydrophobic “a” and “d” residue (Figure 1C). The only one of these mutations that eliminated the interaction with Atg1 was the a/d full (Figure 1D). Given the extensive nature of these mutants, it is somewhat surprising that only the a/d full eliminated binding, although this is consistent with the predicted role of the a/d residues in forming the hydrophobic core of a coiled-coil interaction.

To narrow down the critical residues further, we made a series of double mutants where the “a” and “d” residues within a single heptad repeat were both changed to glutamate (Figure 1C). Of those tested, only the a/d-4 mutant, I562E/Y565E, caused a loss of interaction (Figure 1D). We then individually mutated these two residues, I562 or Y565, to glutamate, glutamine, or alanine. Although the I562E mutation was sufficient to disrupt interaction, I562Q and I562A had no effect. In contrast, all Y565 mutations eliminated the interaction with Atg1 suggesting that this is a particularly critical residue (Figure 1D).

To verify the Y2H results, we performed a coimmunoprecipitation (CoIP) experiment with protein A-tagged ATG1 and GFP-tagged Atg11. Atg1-PA was able to robustly precipitate GFP-Atg11, but not GFP-Atg11ΔCC2. It showed only minimal ability to precipitate GFP-Atg11^Y565E^ (Figures 1E,F) or GFP-Atg11^I562E^ (Supplementary Figures S1A,B) further demonstrating the importance of these residues to the Atg1-Atg11 interaction

### Isoleucine 562 and Tyrosine 565 Are Also Critical for the Interaction of Atg11 With Atg9 and Itself

**FIGURE 2:**
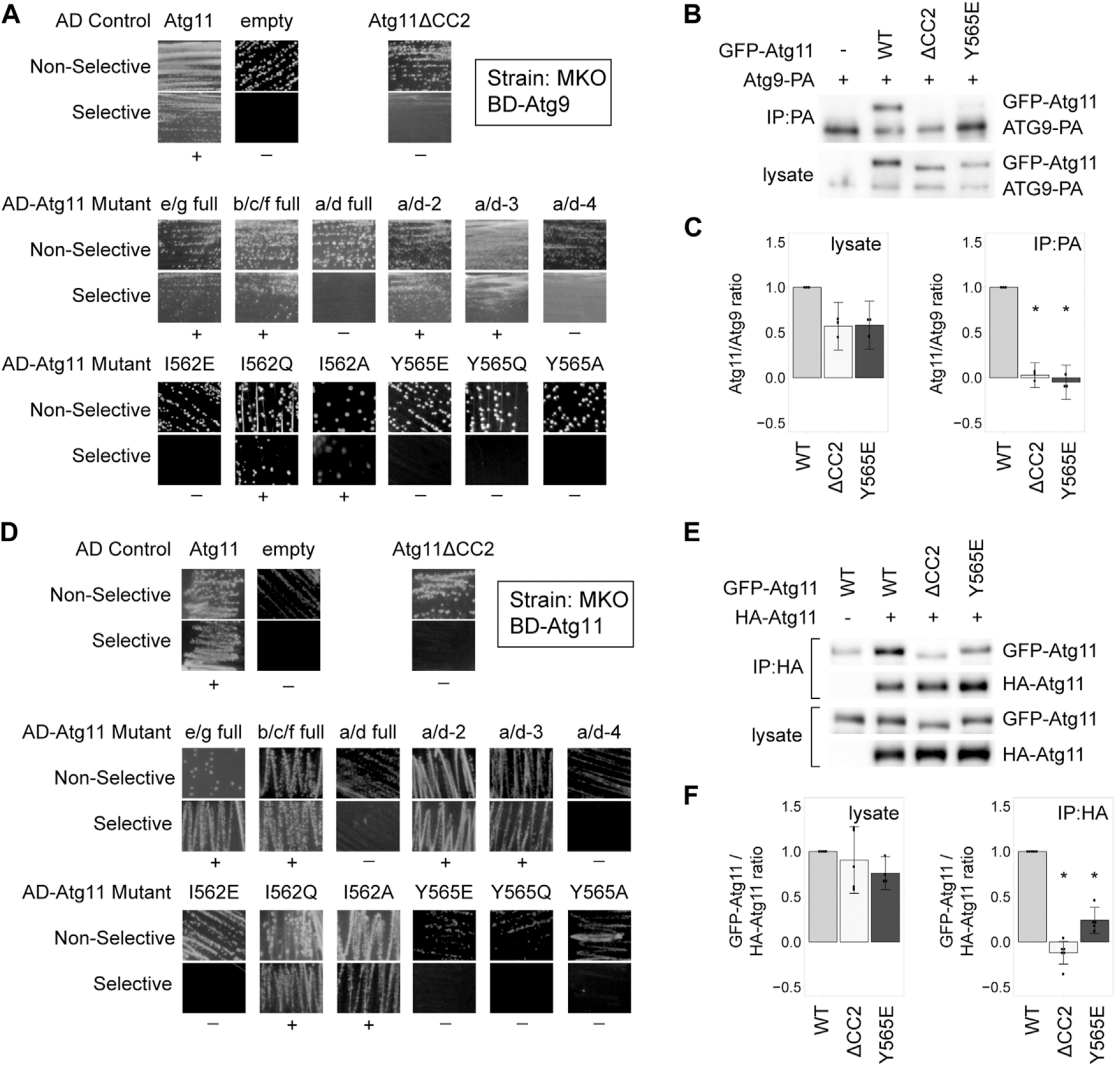
Tyrosine 565 is also critical for the interaction of Atg11 with Atg9 and itself. **(A,D)** Y2H results of BD-Atg9 **(A)** or BD-Atg11 **(D)** with AD-Atg11 mutants in the multiple knockout (MKO) strain. Non-selective media is SMD -ura, -leu, while selective media is either SMD -ura, -leu, -his + 1 mM 3AT **(A)** or SMD -ura -leu -ade -his **(B)**. “+” indicates growth and “−” indicates no growth on selective media in multiple replicates. Examples of full, uncropped Y2H plates are in Supplementary Figure S5B and Supplementary Figure S6A. **(B,E)** CoIP results of GFP-Atg11 mutants with Atg9-PA (**B**) or Atg11-HA **(E)**. Proteins were overexpressed with a *CUP1* promoter in an *atg11∆* strain. **(C,F)** Quantification of CoIP results from ≥ 3 independent replicates, with the background from the negative control subtracted, each normalized to its respective WT sample. Errors bars are 95% CI; * = *p* <0.05 vs WT. Uncropped images of the CoIP blots are in Supplementary Figures S7B,C.

The Atg11 CC2 region has also been reported to be necessary for the interaction with Atg9 (He et al., 2006). To determine which residues were indispensable for this interaction, we tested the same AD-Atg11 mutants with BD-Atg9. Once again, we found that the only residues whose mutation abolished the interaction were I562 and Y565, with Y565 being the most critical (Figure 2A). CoIP data likewise showed that the Y565E mutation led to a complete loss of the Atg9-Atg11 interaction, just like the deletion of the entire CC2 domain (Figures 2B,C), while I562E caused ∼70% loss of interaction (Supplementary Figures S1C,D).

Atg11 also self-interacts, forming a putative dimer, and this interaction has also been shown to require CC2 (Yorimitsu and Klionsky, 2005; Suzuki and Noda, 2018). Therefore, we tested our AD-Atg11 mutants against BD-Atg11. This gave essentially the same results as the Atg1 and Atg9 screens, with the I562E, Y565E, Y565Q, and Y565A mutants disrupting the interaction (Figure 2D). Throughout this screen we observed a certain background of mutants that appeared to retain interaction; approximately 20% of individual transformants from each of the non-interacting mutants retained a robust ability to grow on selective media, even though 80% showed no growth (Supplementary Table S4). This effect was not seen when BD-Atg1∆K or BD-Atg9 were used with the screen, nor was it seen when BD-Atg11 was paired with an empty AD vector. We therefore suspect that these may be revertants resulting from the yeast’s ability to use the unmutated copy of ATG11 in the BD vector to repair the mutated ATG11 present in the AD vector.

To verify these results we performed a CoIP using HA-tagged Atg11, GFP-tagged Atg11, and GFP-tagged Atg11 mutants (Figures 2E,F and Supplementary Figures S1E,F). Under CoIP conditions, both the Y565E mutation and the I562E mutation in GFP-Atg11 led to a >75% loss of interaction.

Overall, the residues in CC2 required for Atg11’s self-interaction are the same as those required for the interaction with Atg1 and Atg9, although Atg11’s self-interaction may be somewhat more resistant to disruption by point mutation.

### Effect of CC2 Mutations on Overall Stability of Atg11

**FIGURE 3:**
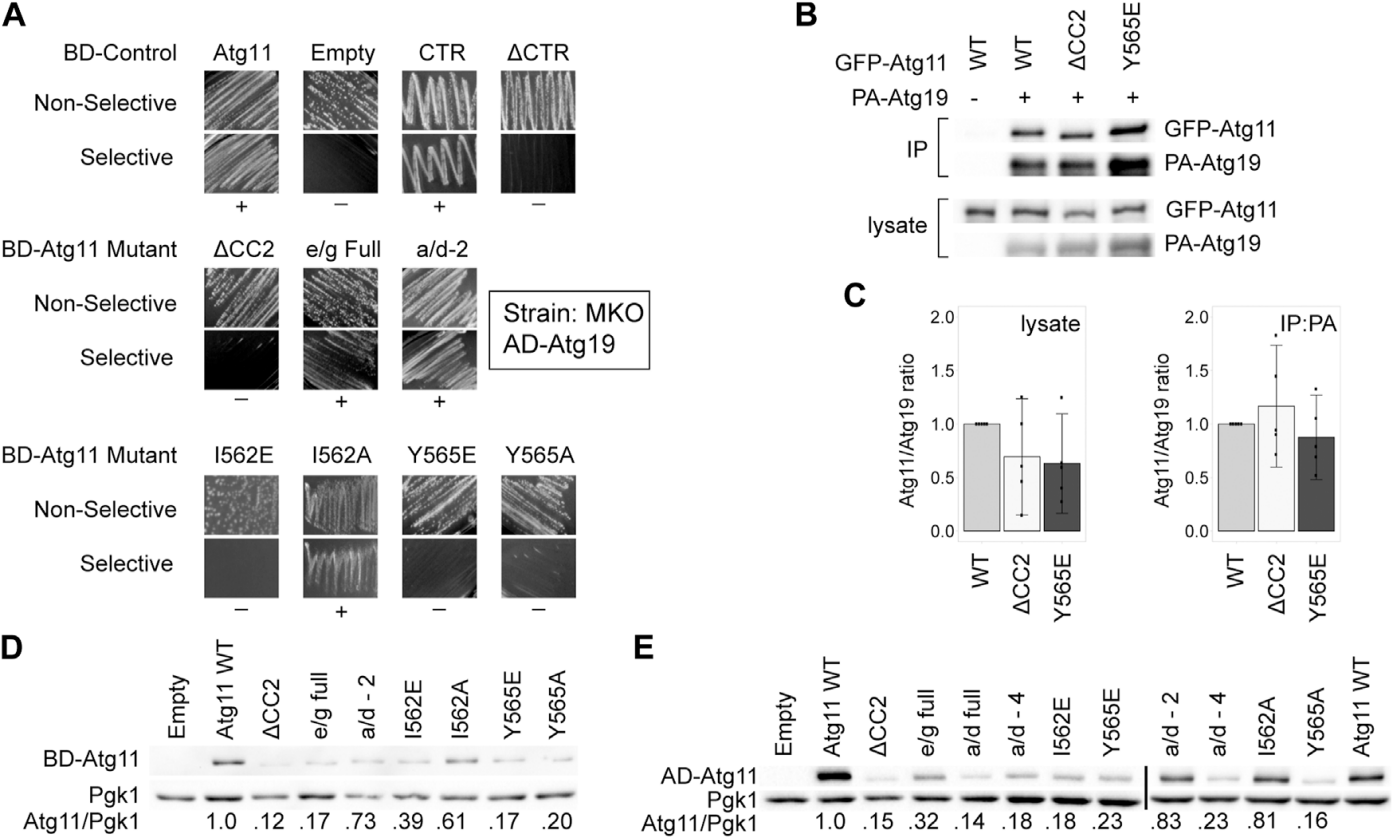
Mutations in CC2 can partially destabilize Atg11 under some conditions. The C-terminus of Atg11 (CTR, a.a. 854-1178) is sufficient for binding to Atg19 in a Y2H assay, yet full length BD-Atg11 with mutations to Y565 loses this interaction and is partially destabilized. **(A)** Y2H results of BD-Atg11 mutants with AD-Atg19 in the MKO strain. CTR = Atg11Δ^1–853^, ΔCTR = Atg11Δ^859–1178^, ΔCC2 = Atg11Δ^536–576^. Other mutants as in Figure 1. Non-selective media is SMD -ura, -leu, while selective media is SMD -ura, -leu, -ade. “+” indicates growth and “−” indicates no growth on selective media in multiple replicates. Examples of full, uncropped Y2H screen are in Supplementary Figure S6B. **(B)** CoIP results of GFP-Atg11 mutants with PA-Atg19. Proteins were overexpressed with a *CUP1* promoter in an *atg11∆* strain. **(C)** Quantification of CoIP results from five independent replicates, with the background from the negative control subtracted, each normalized to the WT lane. Errors bars are 95% CI. **(D,E)** Western blot results showing the stability of the Atg11 mutant constructs in the MKO strains used for the Y2H assays. **(D)** Yeast expressing various BD-Atg11 mutants were blotted with antibodies recognizing the BD tag. **(E)** Yeast expressing BD-Atg1ΔK and various AD-Atg11 mutants were blotted with antibodies recognizing the AD tag. Pgk1 was used as a loading control. Quantifications shown in **(D,E)** are the average of four independent replicates. Uncropped images of the western blots are in Supplementary Figure S7D and Supplementary Figures S8A,B.

Since three independent interactions were disrupted by the same mutations to Atg11, we wondered if these mutations had completely disrupted the structure of the entire Atg11 protein. We tested this by determining the ability of these Atg11 mutants to interact by Y2H with Atg19, which is known to bind in the C-terminal region of Atg11 (Yorimitsu and Klionsky, 2005). This region has direct homology to the FIP200 “claw” region, and is thought to form an autonomously folded domain (Turco et al., 2019), and thus should not be affected by mutations in CC2 unless these are disrupting the entire protein. To avoid the autoactivation seen with BD-Atg19, we transferred Atg11 and its mutants to the BD vector and tested them against AD-11. To our surprise, deletion of the CC2 region caused a loss of interaction with Atg19 in our MKO Y2H assay, even though the deletion of the entire N-terminal portion of Atg11, including CC2 and CC3, still gave robust interaction in this assay (Figure 3A). Similarly, mutations in CC2 such as Y565E that caused a loss of interaction with Atg1, Atg9, and Atg11, also caused a loss of interaction with Atg19 as tested by this assay. However, when we tested the Atg11-Atg19 interaction by CoIP using GFP-Atg11 and PA-Atg19, we found that the interaction was not disrupted – neither Atg11ΔCC2 nor Atg11^Y565E^ lost any ability to coprecipitate with Atg19 (Figures 3B,C), even though they no longer coprecipitated with Atg1, Atg9, or Atg11.

We next tested the expression levels of these Atg11 mutants in the MKO yeast-hybrid strain by immunoblotting with anti-BD antibodies. As can be seen in Figure 3D, deletion of the CC2 domain and many of the mutations to it caused a significant loss of BD-Atg11 expression. Identical results were seen when the immunoblotting for the AD-Atg11 mutants used for the Y2H experiments with Atg1, Atg9, and Atg11 (Figure 3E). Interestingly, only a minor loss of expression was observed in the GFP-Atg11ΔCC2 and GFP-Atg11 point mutants used for the CoIP assays (Figure 1F, Figures 2C,F, and Figure 3C), suggesting that the effect of the mutations on stability are influenced by factors such as protein tag or growth conditions. Similarly, mutations to the CC2 domain had no effect on the expression, stability, or solubility of the CC2-3 region of Atg11 when it was heterologously expressed in *E. coli* (Supplementary Figure S2). This suggests that mutations to the CC2 may have an effect on the structure of Atg11 that is dependent on the context of the entire protein, or the native yeast cellular environment.

A previous study (Yorimitsu and Klionsky, 2005) reported that Atg11ΔCC2 retained interaction with Atg19, as measured both by CoIP and Y2H. Although our CoIP results are consistent with these results, in our hands the Y2H assay did show a loss of interaction when CC2 was deleted or mutated. One difference between our assay and theirs is that ours was conducted in the MKO strain, where other Atg proteins are absent, whereas theirs was done in a WT Y2H strain, PJ-694a. Therefore, we repeated the Y2H assay with BD-Atg11 mutants and AD-Atg19 using the PJ-694a strain (Supplementary Figure S3A). However, our results in this strain were identical to our results in the MKO Y2H strain, so this does not explain the difference.

Our CoIP assay was performed on log-phase cells growing in liquid culture, whereas the Y2H assay measured growth on plates. We hypothesized that the growth conditions of the yeast might affect the results of the Y2H assay, perhaps by modulating the stability of Atg11. However, a quantitative Y2H assay performed in log-phase liquid cultures expressing BD-Atg11 mutants and AD-Atg19 gave the same results as were seen with the plate-based Y2H (Supplementary Figure S3B). Therefore, we concluded that growth conditions alone do not explain the difference between the Y2H and the CoIP results for the Atg11/Atg19 interaction.

Overall, these results suggest that the Y565E and I562E mutations have an effect on the overall structure and stability of Atg11, but a stronger effect on the interaction of Atg11 with Atg1, Atg9, and its self-interaction. This effect may be due to the direct participation of these residues in binding, or it may be that they are critical for stabilizing an entire region of Atg11 (such as perhaps the N-terminal half) that is needed for these interactions.

### The I569E Mutation Also Blocks the Function of the CC2 Region

After we had completed our directed Y2H screen of the CC2 region, but while verification of mutational effects was still underway, a study was published from another group that used random mutagenesis of the CC2 region combined with Y2H screening to identify a mutation that caused a loss of interaction with Atg9 (Yao et al., 2020). The mutation identified, I569E, is strikingly similar to the I562E and Y565E mutations that were identified here. In fact, I569 is the very next hydrophobic “a/d” position in the heptad repeat pattern after Y565. The authors showed that the I569E mutation blocked the interaction of Atg11 with Atg9 and its function in glucose starvation-induced autophagy. Given its proximity to Y565, we suspected I569E might also block Atg11 dimerization and its interaction with Atg1, which were not tested in that paper. To confirm, we performed Y2H and CoIP assays with AD-Atg11^I569E^ and found that the I569E mutation behaved the same as the I562E and Y565E mutations, blocking the interaction with Atg1 and Atg11, and reducing the expression levels of AD-Atg11 in the MKO strain (Supplementary Figure S4). Therefore, I569, like I562 and Y565, is required not only for the interaction of Atg11 with Atg9, but also its interaction with Atg1 and Atg11’s self-interaction.

### Mutations That Disrupt Atg11’s Interactions Also Block Selective Autophagy

Atg11 is required for the cytoplasm-to-vacuole targeting (cvt) pathway, a type of selective autophagy that delivers the protease Ape1 to the vacuole where it is activated by proteolytic cleavage. Monitoring the conversion of cytosolic prApe1 to cleave vacuole mApe1 is a standard assay for the functionality of the cvt pathway and Atg11 (Guimaraes et al., 2015).

**FIGURE 4:**
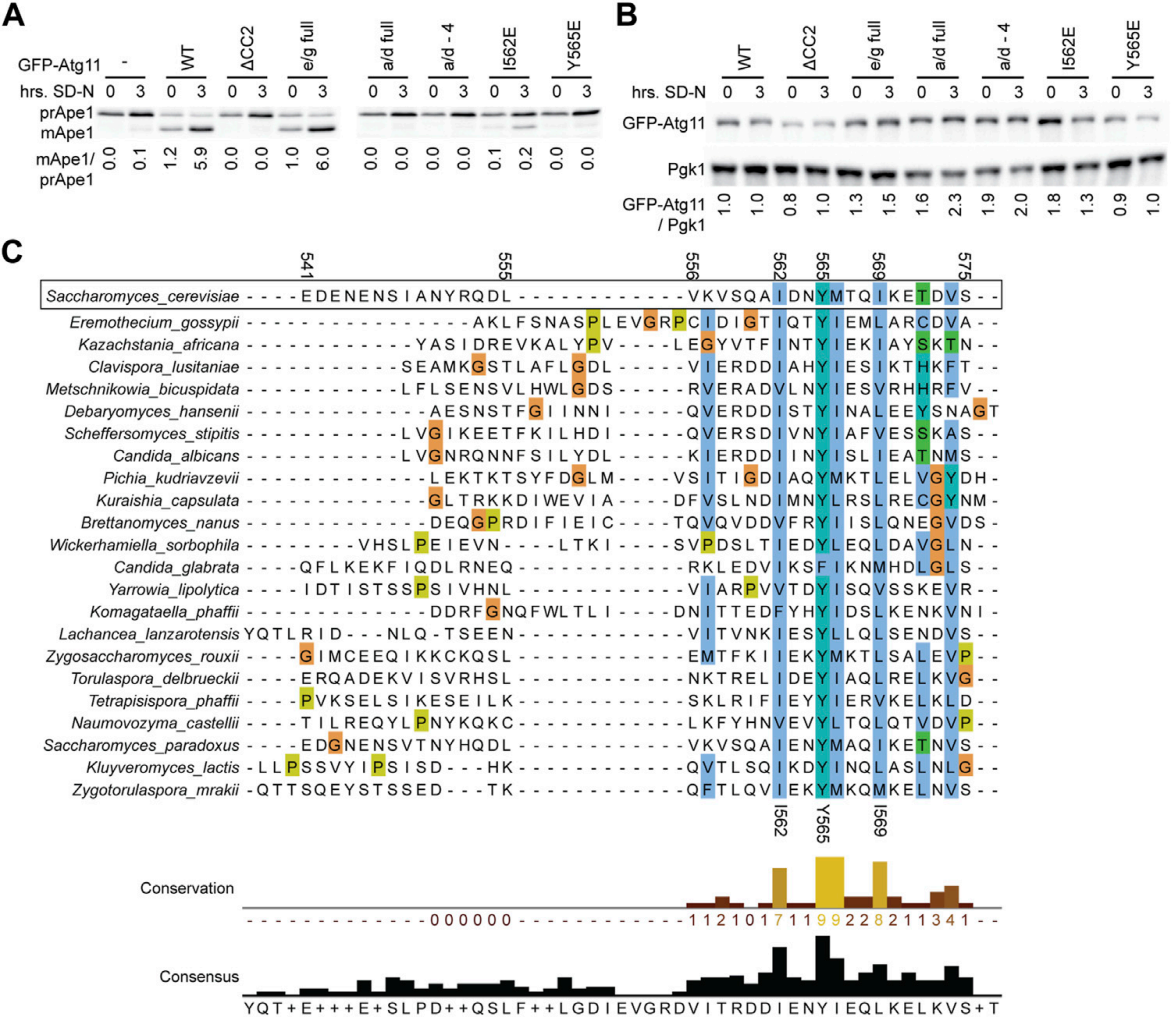
Mutations in CC2 render Atg11 nonfunctional. **(A)** The functionality of Atg11 was measured via the Ape1 processing assay; yeast pellets from an *atg11Δ* strain expressing GFP-Atg11 and its mutants from a *CUP1* promoter were harvested in nutrient rich conditions (0) or after 3 h of nitrogen starvation (3) and blotted against Ape1. The ratio of mature, cleaved Ape1 (mApe) to prApe1 indicates the functionality of Atg11 in the cvt pathway. **(B)** The expression levels of Cu-GFP-Atg11 were assessed in the same samples as panel A by blotting against the GFP tag; Pgk1 was used as a loading control. Quantifications are the average of three blots of independent biological replicates. Uncropped images of the western blots are in Supplementary Figures S8C,D. **(C)** Alignment of the Atg11CC2 region across diverse budding yeast species, color coded by conservation and amino acid class (ClustalX). Residue numbers are indicated based on the *S. cerevisiae* sequence.

Maturation of Ape1 was assessed under both growing conditions and after 3 h of nitrogen starvation (which boosts expression and trafficking of Ape1) in *atg11Δ* cells expressing GFP-Atg11 mutants (Figure 4A). GFP-Atg11 was overexpressed under the CuPI promoter in this assay to ensure sufficient expression levels of the mutants, and the expression of all constructs was verified by western blotting (Figure 4B). The Y565E mutation was sufficient to generate a complete block in Ape1 processing, reducing the amount of mApe1 to below background levels. In contrast, the e/g-full mutation, which despite its extensive nature does not block Atg11’s dimerization or its interaction with Atg1 or Atg9, showed no defect in the cvt pathway, supporting mApe1 generation to the same extent as WT Atg11. Interestingly, although the I562E mutation caused a >80% reduction in the amount of Ape1 processed, it did not cause a complete block like Y565E, consistent with the idea that I562 is less critical to the function of the CC2 region than Y565.

### Y565 Is Highly Conserved Across Budding Yeast Species

As an orthogonal method to assess the importance of the residues we had identified by our systematic mutagenesis, we aligned the CC2 region of *S. Cerevisiae* Atg11 with the corresponding region of Atg11 homologues from 22 other species of budding yeast (order Saccharomycetales). The first half of the CC2 showed very little conservation, but the second half showed conservation of some key hydrophobic residues (Figure 4C). The most conserved residue was Y565, which was a tyrosine in 21 out of the 22 species, and a phenylalanine in the remaining one (*C. glabrata*). This speaks not only to the importance of the aromatic ring, but also suggests that the hydroxyl group may play a role in stabilizing the structure. I562 was the second most conserved residue, followed by I569 and M566. All of these positions were invariably hydrophobic across all species analyzed and presumably help to anchor the structure of this key region.

## Discussion

Here we identify tyrosine 565 in the 4^th^ repeat of CC2 as a key residue whose mutation blocks Atg11’s interaction with Atg1 and Atg9, which also largely disrupts its self-interaction. Tyrosine 565 is a series of four residues (I562, Y565, M566, and I569) whose hydrophobic nature is conserved across multiple yeast species, and which may form the hydrophobic core of a coiled-coil structure critical for Atg11’s structure, interactions, and function. M566 is actually predicted to fall in a hydrophilic “e” position in the heptad repeat, and therefore was not identified in our screen because in the “e/g full” mutant it was changed to alanine, which actually preserved its hydrophobic nature. The other three residues are in the hydrophobic a and d positions, and the individual mutation of any of these residues to a charged, hydrophilic residue (glutamate) render Atg11 non-functional. Y565 is even more crucial than I562 as evidenced by the fact that I562 can tolerate more conservative mutations, such as mutation to a smaller hydrophobic (alanine) or hydrophilic uncharged (glutamine) residue, whereas Y565 cannot. The large aromatic ring of the tyrosine may play a particularly important stabilizing role for Atg11 CC2, consistent with the fact that it is completely conserved as an aromatic across all 23 species of yeast analyzed.

The I569E mutation was recently identified by the Yi lab, who discovered that it interfered with Atg11’s interaction with Atg9 and blocked both the cvt pathway and glucose starvation-induced autophagy (Yao et al., 2020). Our data confirms the importance of I569 for Atg11’s interaction with Atg9 but shows that it is also important for Atg11’s interaction with Atg1. Therefore, the phenotype of the Atg11 I569E mutation should be interpreted with caution, as it likely results not only from the loss of the Atg9-Atg11 interaction but also from the loss of the Atg1-Atg11 interaction and possibly Atg11 dimerization.

A recent study from the Ragusa lab reported that Atg11’s CC3 domain forms a parallel dimer with a well-ordered core but with significant flexibility on either end, likely due to defects in the hydrophobic heptad repeat sequence in these regions (Margolis et al., 2020). There is a similar lack of hydrophobic residues in the expected positions at either end of the CC2 domain, and a high proportion of disorder promoting residues, suggesting that CC2 may be quite flexible. However, despite this predicted flexibility it is very important to the overall structure of Atg11. This flexibility and the relatively poor adherence to the heptad repeat pattern may explain why this domain can be disrupted by a single point mutation, whereas often coiled-coil interactions are more stable and require multiple mutations to disrupt. Interestingly; however, double mutations of hydrophobic residues in the N-terminal portion of the CC2 domain (I548E/Y551E, “a/d-2”, L555E/V558E, “a/d-3) did not cause any loss of Atg11 interaction. Therefore, it is not just a matter of CC2 being on the edge of stability and disrupted by any mutation to its hydrophobic core; instead, there is a particular importance of the region stabilized by I562, Y565, and I569 for the function and interactions of Atg11.

Additional evidence of the importance of these residues includes that under some conditions their mutants lead to a partial destabilization of Atg11. The extent of this destabilization appeared to vary based on tag and growth condition, and no destabilization was seen when a fragment containing the CC2-CC3 region was heterologously expressed in *E. coli*, underscoring the subtlety of this effect. It was, nevertheless, reproducible in each given condition, and may partially account for the different results obtained for the Atg11-Atg19 interaction with the Y2H assay vs CoIP. The strains and constructs used for Y2H showed an approximate 3-fold greater reduction in the expression levels of the ΔCC2 and Y565E mutants than did those used for the CoIP (Figures 3C,D). However, this is not a complete explanation for the loss of growth on the selective Y2H plates in these mutants since the e/g full mutant, which showed an equivalent reduction in expression levels, still retained the ability to grow. Interestingly, all mutants retained interaction with Atg19 as shown by CoIP. This demonstrates that these mutations did not cause the entire Atg11 protein to misfold. However, since the C-terminus is expected to form an autonomously folding domain, this does not rule out the possibility that these mutations could have caused the entire N-terminal region, possibly even including CC3, to misfold.

The CC2 region appears to play a particularly important role in the dimerization of *S. cerevisiae* Atg11. In fission yeast (*S. pombe*), residues 546-583 of Atg11 mediate its homodimerization, while residues 522-552 bind to Atg1 (Pan et al., 2020) . These regions align to a.a. 671-731 in *S. cerevisiae* Atg11, which overlaps CC3. Interestingly, while Yorimitsu and Klionsky reported that deletion of either CC2 or CC3 disrupted Atg11’s self-interaction in a Y2H screen, the Ragusa lab more recently showed via CoIP that deletion of CC3 does not block Atg11’s ability to interact with itself (Yorimitsu and Klionsky, 2005; Margolis et al., 2020). In contrast, deletion of the CC2 region does block Atg11 self-interaction (Yorimitsu and Klionsky, 2005), as does mutation of Y565 within the CC2. This suggests that for budding yeast, CC2, not CC3, is the region most important for dimerization, although other regions likely also contribute.

Mutation of Y565 also blocks the interaction of Atg11 with its partners Atg1 and Atg9, and this effect seems even stronger than the disruption of Atg11 self-interaction. CC2 was previously known to be important for these interactions, although they also require other regions (CC3 for Atg1 and the N-terminus for Atg9) (Yorimitsu and Klionsky, 2005; He et al., 2006). The fact that both of these interactions as well as the Atg11 self-interaction depend on the exact same residue may suggest a shared binding site. Alternately, it may suggest that Y565 is the linchpin necessary for the folding of a larger region, perhaps the entire N-terminus, that encompasses the binding sites for both Atg1 and Atg9.

Recently, a predicted structure of Atg11 became available as part of a large number of structural models made by the neural-network based folding algorithm AlphaFold (Jumper et al., 2021). This model has not been validated, and it only models a single copy of Atg11, not the known homodimeric structure. Nevertheless, the model aligns well with the experimentally determined structure for the CC3 region of Atg11. In this model, CC2 (a.a. 541-575) is predicted with 70–90% confidence to form a short alpha helix with disordered ends and to interact with two other helices, a.a. 461–481 and a.a. 575–590. It also has a face free for additional potential interactions. I562, Y565, and I569 (though not M566) are all facing the interior of the protein in this model, part of a hydrophobic cluster that also includes L464 and L582 from the other helices. In addition, the phenolic hydroxyl of Y565 is predicted to hydrogen bond with the backbone carbonyl of P457. This prediction is intriguing in light of the fact that 22 out of 23 homologues of Atg11 in other yeast species maintained a tyrosine at this position. If this model is accurate, it seems reasonable that mutations to these residues, in particular Y565, could lead to the destabilization of this structure and thus loss of a binding surface for Atg1, Atg9 and Atg11 dimerization.

Although our experiments allowed us to identify specific residues in the CC2 region that are essential for Atg11’s interactions, they have unfortunately not allowed us to determine whether these residues are directly involved in binding, are structurally important to Atg11, or both. Determining this will require biophysical characterization of purified WT and mutant Atg11. Unfortunately, these experiments could not be carried out because a well behaved Atg11 fragment could not be expressed and purified. A GST-tagged CC2-3 fragment expressed well and gave moderate yield after purification but was not suitable for characterization; it formed a very large complex, possibly a soluble aggregate, and precipitated upon proteolytic removal of the GST tag. The CC2 region may require the remainder of the N-terminus in order to adopt a native conformation; however, when we attempted expression of larger constructs that included the N-terminus in *E. coli* we saw very little expression and significant degradation, possibly because the N-terminus is intrinsically disordered. Ultimately, further experiments and direct structural information will be necessary to fully understand the critical role that these residues are playing in Atg11’s structure, interactions, and overall function.

## Data Availability

The original contributions presented in the study are included in the article/Supplementary Material, further inquiries can be directed to the corresponding author.

## Abbreviations

AIC, autophagy initiation complex; Atg, autophagy related; CC, coiled-coil; CTR, c-terminal region; CoIP, coimmunoprecipitation; LIC, ligation independent cloning; MKO, multiple knock-out; PMSF, phenylmethylsulfonyl fluoride; TCA, trichloroacetic acid; Y2H, yeast-2-hybrid.
