# Supplementary material for: Mapping Critical Residues in ATG11’s Coiled-Coil 2 Domain that Block Multiple Interactions and Disrupt Selective Autophagy
Source: Front Cell Dev Biol. 2022 Jan 18;9:775364. doi: 10.3389/fcell.2021.775364 (PMC8805157; doi:10.3389/fcell.2021.775364)
Supplement: Supplementary file 1 [file DataSheet1.pdf]

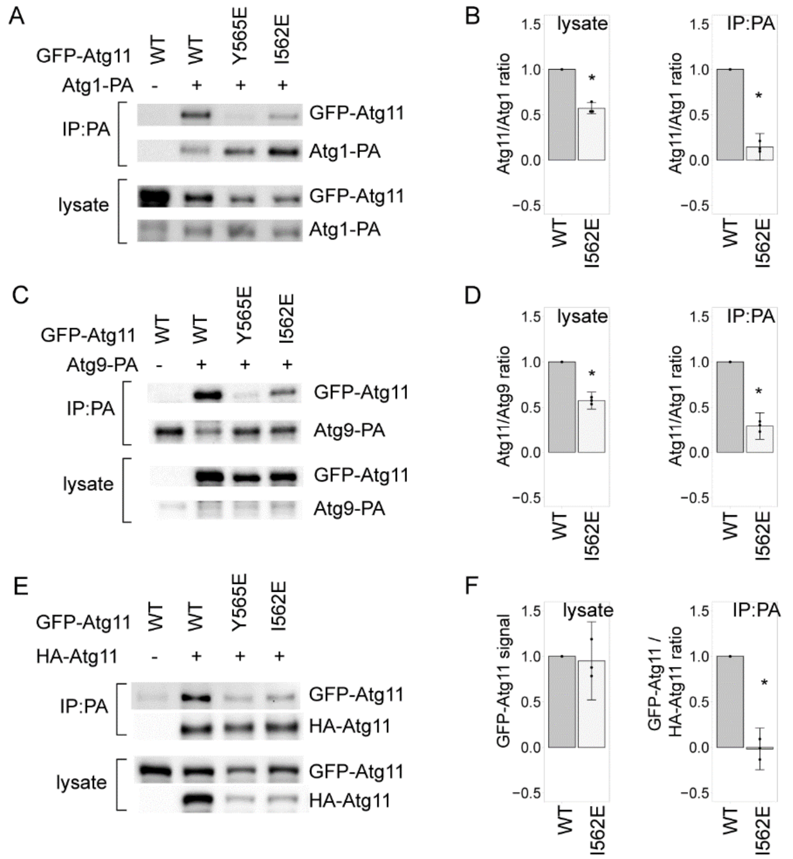

Supplementary Figure 1: The I562E mutation, like Y565E, disrupts the interaction of Atg11 with Atg1, Atg9, and itself as measured by CoIP. CoIP assays showing the ability of (**A**) endogenously driven Atg1-PA, (**C**) overexpressed Atg9-PA, or (**D**) overexpressed HA-Atg11 to precipitate overexpressed GFP-Atg11 and its mutants, including I562E; **(B,D,F)** Quantification of CoIP results from **(A,C,E)**: three I562E replicates from a single experiment, with the background from the negative control subtracted, each normalized to the single WT sample. Note that for figure F, the GFP-Atg11 signal was not adjusted to the HA-Atg11 signal because the latter was overloaded in the WT lane and so the quantification was more accurate without this adjustment. Errors bars are 95% CI; * = $p < 0.05$ vs WT. Full, uncropped CoIP blots are available in **Supplementary Figure 9A,B,C**.

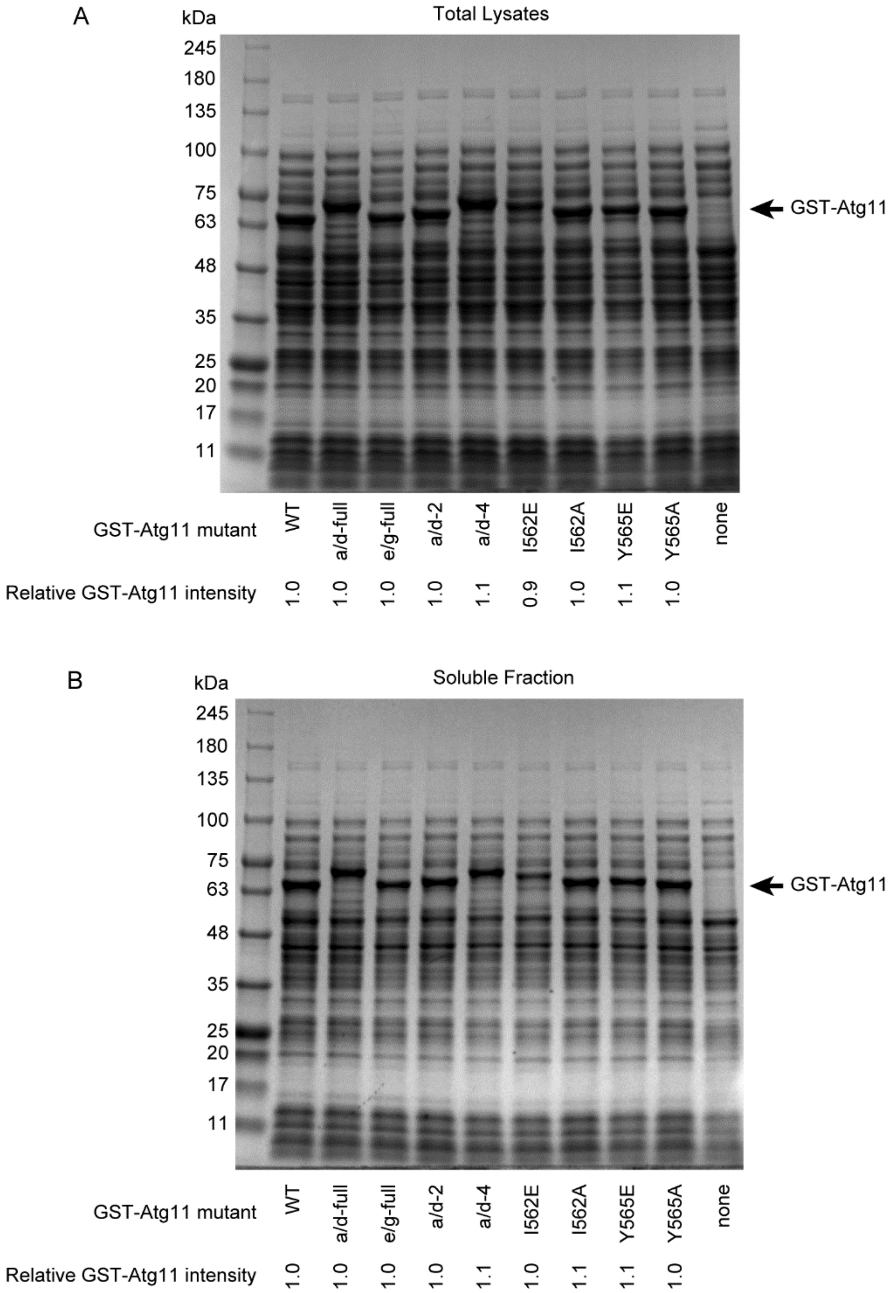

Supplementary Figure 2: Mutations to the CC2 region of ATG11 do not fundamentally destabilize it. GST-ATG11CC2-3 (a.a. 537-853) with various CC2 mutations was expressed in *E. coli*. BL21 cells containing pMCSG10-ATG11CC2-3 were autoinduced, lysed by sonication, and a soluble fraction generated by centrifugation at 16,000xg. **(A)** Total lysates and **(B)** the soluble fraction were each analyzed by SDS-PAGE and Coomassie staining to determine the expression levels and solubility of each mutant. Quantification is the intensity of the GST-Atg11 band divided by the average of the other bands in that lane, normalized to WT, and averaged over four (A) or two (B) independent replicates.

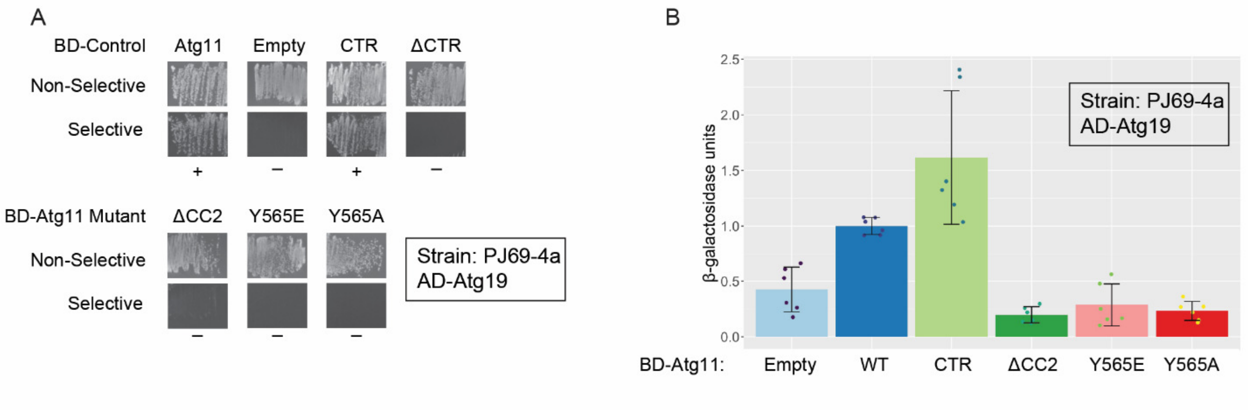

Supplementary Figure 3: The presence of other Atg proteins does not affect the Atg11-Atg19 interaction.

(A) Yeast-2-hybrid results of BD-Atg11 mutants with AD-Atg19 in a wild type yeast-2-hybrid strain (PJ69-4a) that contains all Atg proteins. Non-selective media is SMD -ura, -leu, while selective media is SMD -ura, -leu, -ade. "+" indicates growth and "-" indicates no growth on selective media in multiple replicates. Results were identical to those seen in the multiple knockout strain (Figure 3A). **(B)** Quantitative liquid Y2H results of BD-Atg11 mutants with AD-Atg19 in PJ69-4a. β-galactosidase activity indicates expression from the Gal promoter and thus strength of the interaction. Results match those seen on solid media in panel (A). N=6; error bars = SD. Abbreviations as in Figure 3.

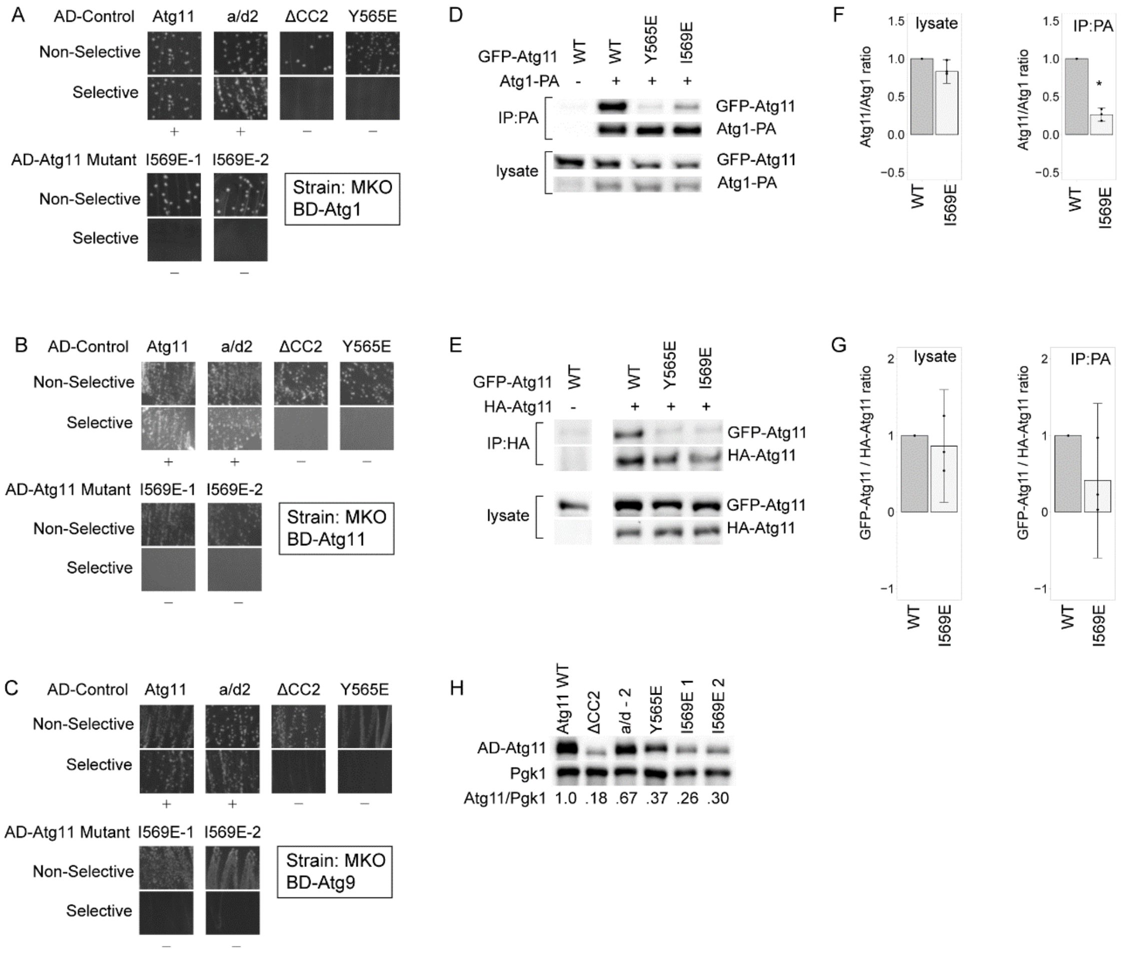

Supplementary Figure 4: The I569E mutation has similar effects as Y565E.
(A-C) Atg11 I569E, like Atg11 Y565E, loses interaction with not only Atg9 (**A**) but also with Atg1 **(B)**, and Atg11 (**C**), as measured by the Y2H assay in the MKO strain. Assay conditions are as in Figures 1-3; 1569E-1 and I569E-2 are two independent clones of the Atg11 I569E mutant. **(D-E)** CoIP results of GFP-Atg11 mutants, including I569E, with Atg1-PA (**B**) or Atg11-HA (**E**). Proteins were overexpressed with a *CUP1* promoter in an *atg11*Δ strain. **(F)** Quantification of CoIP results from (**D**): three I569E replicates from a single experiment, with the background from the negative control subtracted, each normalized to the single WT sample. (**E**) Quantification of CoIP results from 3 independent replicates, with the background from the negative control subtracted, each normalized to its respective WT sample. Errors bars are 95% CI; * = p <0.05 vs WT. (**H**)The I569E mutation reduces expression of AD-Atg11 to a similar extent as Y565E. MKO yeast expressing two clones of AD-Atg11 I569E or other AD-Atg11 mutants were blotted with antibodies recognizing the AD tag. Quantifications are the average of three blots of independent biological replicates. Full, uncropped CoIP blots are available in **Supplementary Figure 9D,E**.

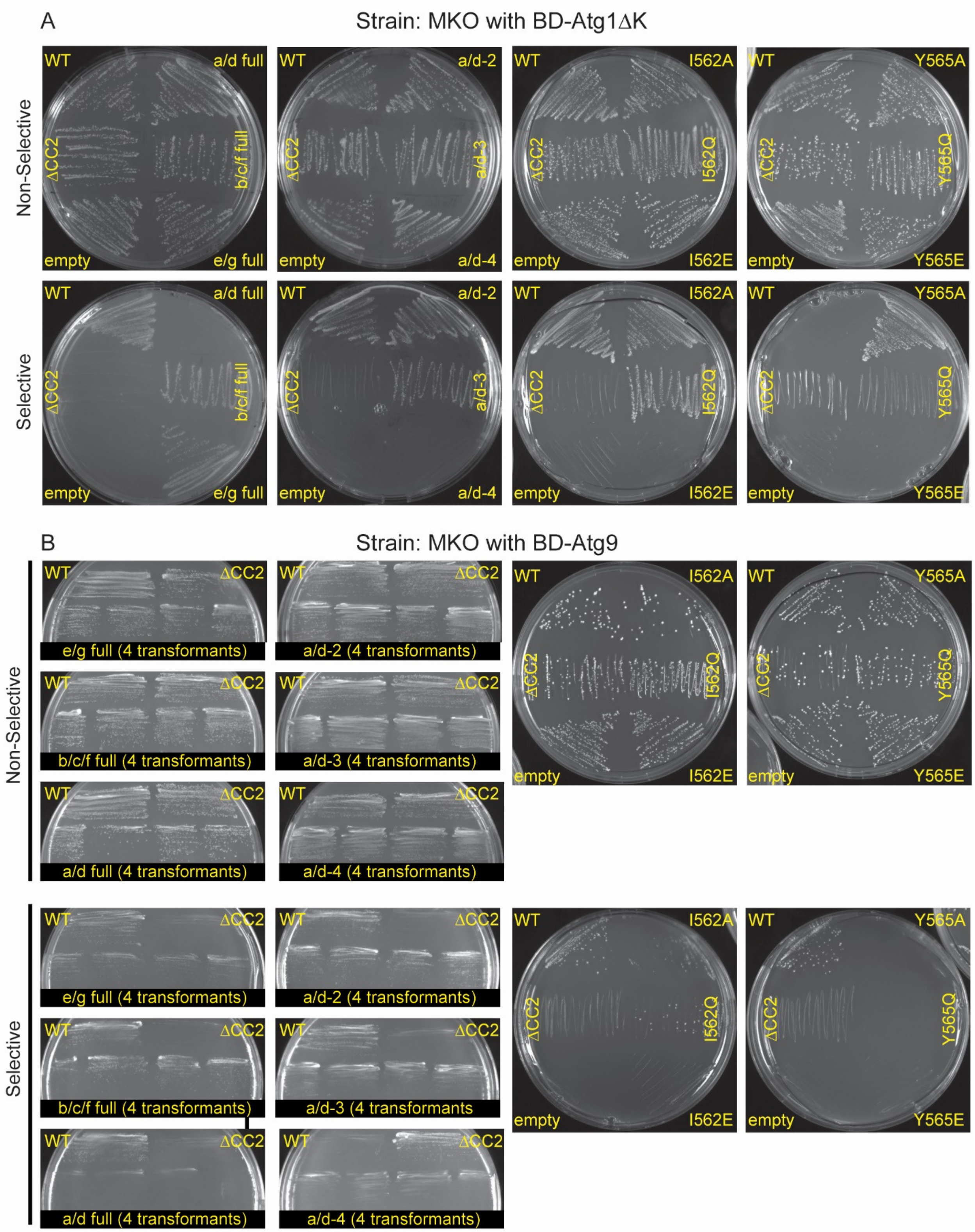

Supplementary Figure 5: Full plate images of Atg1ΔK and Atg9 Y2H screens. MKO yeast containing BD-Atg1ΔK (**A**) or BD-Atg9 (**B**) were transformed with AD-Atg11 mutant plasmids and struck on selective and non-selective plates. Abbreviations as in Figure 1.

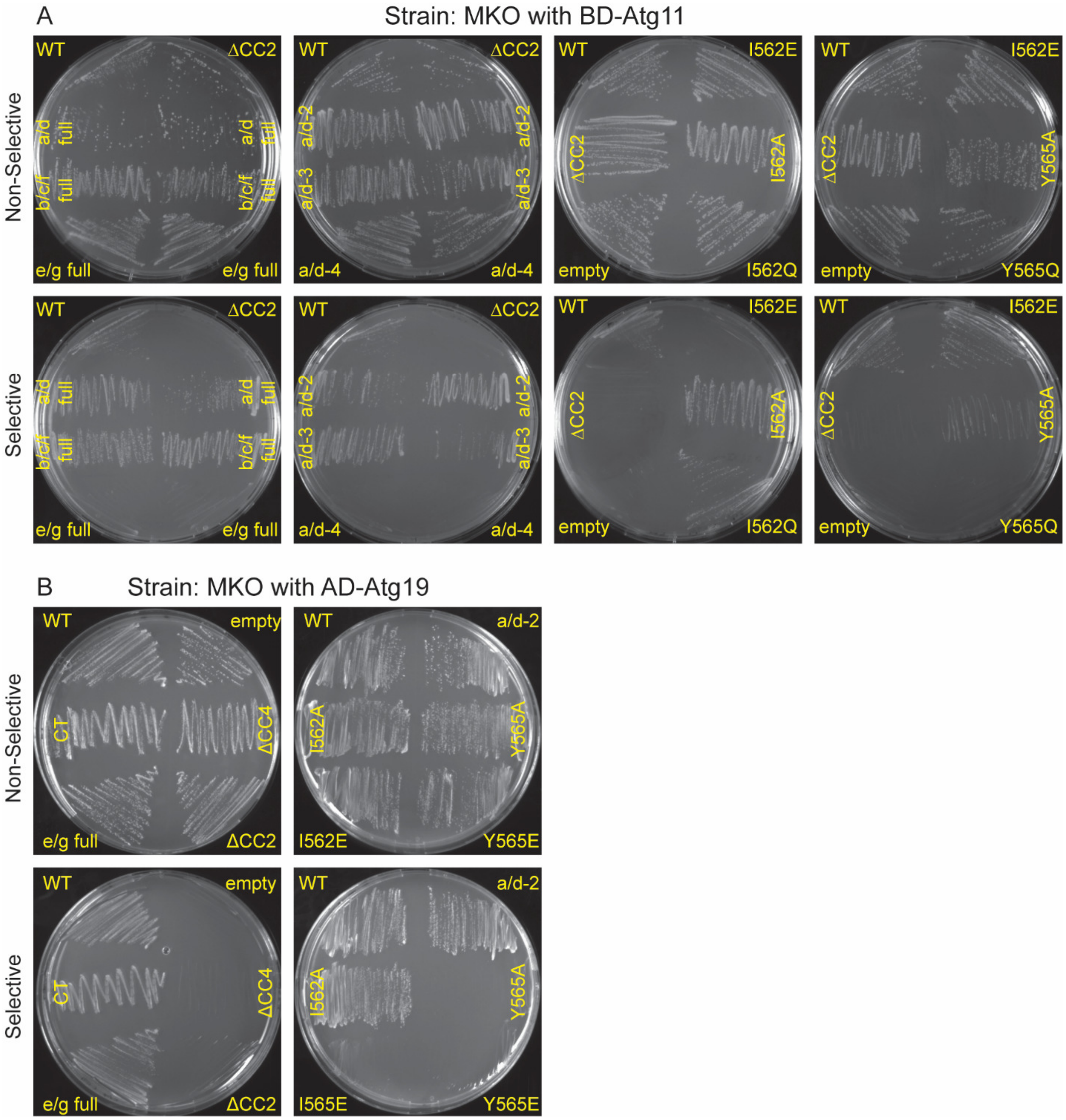

Supplementary Figure 6: Full plate images of Atg11 and Atg19 Y2H screens. **(A)** MKO yeast containing BD-Atg11 were transformed with AD-Atg11 mutant plasmids and struck on selective and non-selective plates. **(B)** MKO yeast were transformed with BD-Atg11 mutant plasmids followed by AD-Atg19 then struck on selective and non-selective plates. Abbreviations as in Figures 1 and 3.

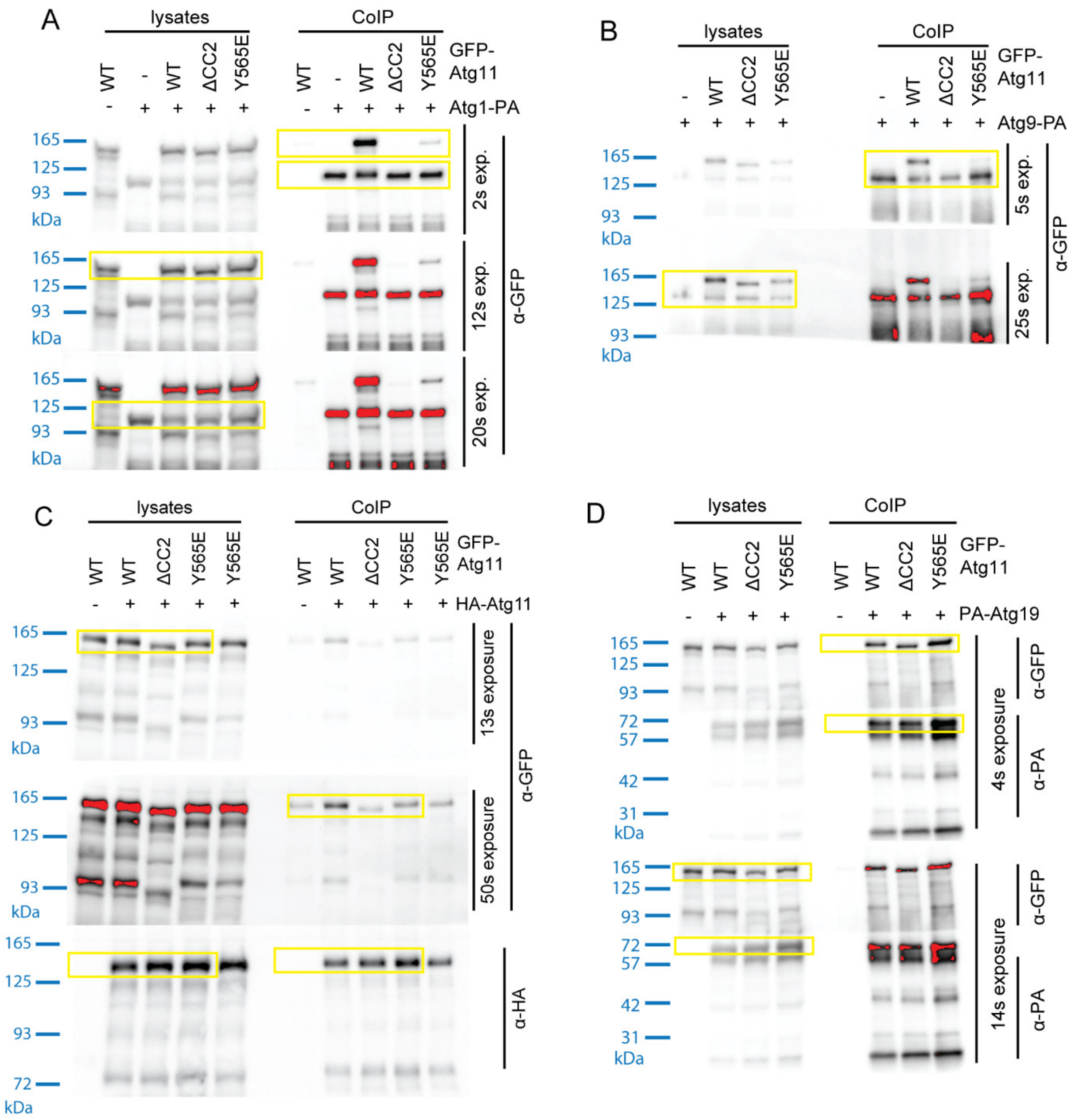

Supplementary Figure 7: Uncropped blot images for CoIPs shown in main figures. (**A**) Atg1-PA / GFP-Atg11 CoIP from Figure 1. (**B**) Atg9-PA / GFP-Atg11 / CoIP from Figure 2. (**C**) HA-Atg11 / GFP-Atg11 CoIP from Figure 2. (**D**) PA-Atg19 / GFP-Atg11 CoIP from Figure 3. All abbreviations as in those figures. Yellow boxes indicate the region of the blot presented in the main figure. Red indicates overexposed bands, which were not quantitated or presented. Positions of molecular weight ladder bands shown in blue.

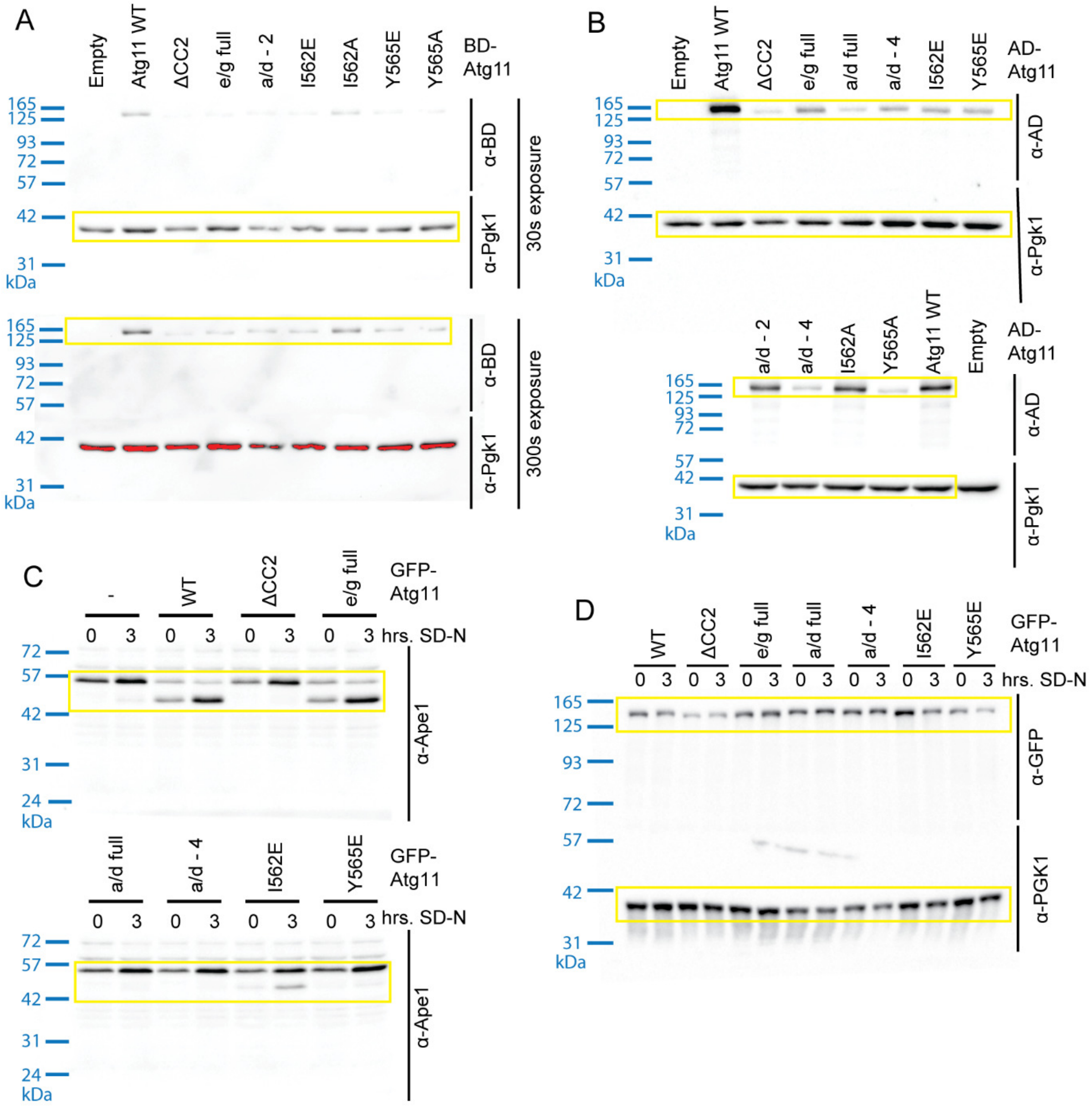

Supplementary Figure 8: Uncropped images for western blots shown in main figures. **(A)** BD-Atg11 quantification from Figure 3. **(B)** AD-Atg11 quantification from Figure 3 **(C)** Ape1 processing assay from Figure 4. **(D)** GFP-Atg11 quantification from Figure 4. All abbreviations as in those figures. Yellow boxes indicate the region of the blot presented in the main figure. Red indicates overexposed bands, which were not quantitated or presented. Positions of molecular weight ladder bands shown in blue.

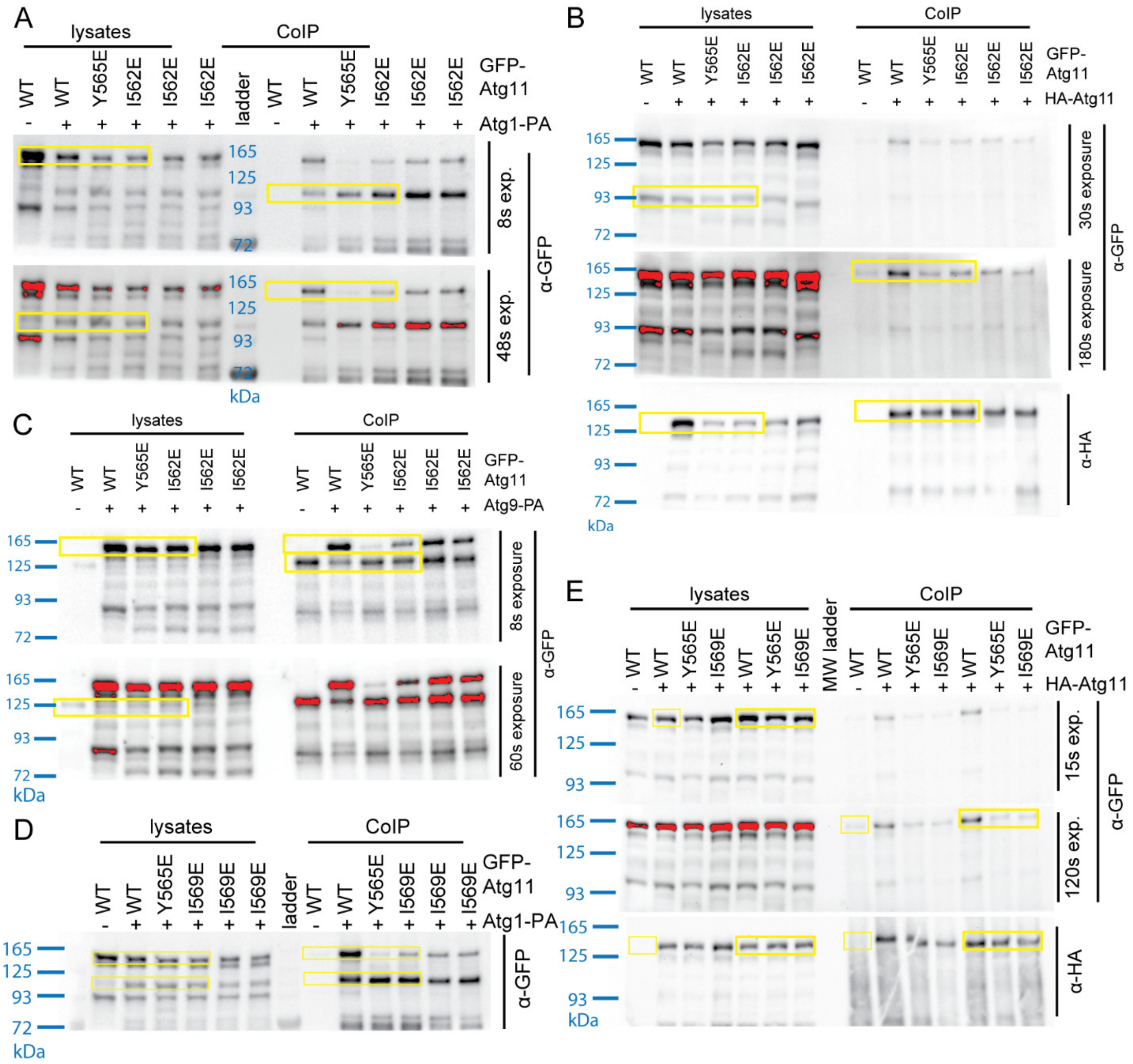

Supplementary Figure 9: Uncropped images for western blots shown in main figures. (**A**) Atg1-PA / GFP-Atg11 I562E CoIP from Supplementary figure 1. (**B**) HA-Atg11 / GFP-Atg11 I562E CoIP from Supplementary figure 1. (**C**) Atg9-PA / GFP-Atg11 I562E CoIP from Supplementary figure 1. (**D**) Atg1-PA / GFP-Atg11 I569E CoIP from Supplementary figure 4. (**E**) HA-Atg11 / GFP-Atg11 I569E CoIP from Supplementary figure 4. All abbreviations as in those figures. Yellow boxes indicate the region of the blot presented in the main figure. Red indicates overexposed bands, which were not quantitated or presented. Positions of molecular weight ladder bands shown in blue.

Supplementary Table 1: Plasmids used in this study

Name	Description	Source
pGAD-C1	Empty Y2H AD vector	(James et al., 1996)
pGAD-ATG19	AD vector with full length ATG19	(Yorimitsu and Klionsky, 2005)
pGAD-ATG11	AD vector with full length WT ATG11	This study
pGAD-atg11ΔCC2	AD vector with atg11$\Delta^{536\text{-}576}$	(Yorimitsu and Klionsky, 2005)
pGAD-atg11CTR	AD vector with atg11$\Delta^{1\text{-}853}$	This study
pGAD-atg11ΔCTR	AD vector with atg11$\Delta^{859\text{-}1178}$	This study
pGAD-atg11 e/g Full	AD vector with atg11$^{E545A/S547A/R552A/D554A/S559A/A561A/M566A/Q568A/D573A/S575A}$	This study
pGAD-atg11 b/c/f Full	AD vector with atg11$^{D542A/E543A/N546A/A549A/N550A/Q553A/V556A/K557A/Q560A/D563A/N564A/T567A/K570A/E571A/V574A}$	This study
pGAD-atg11 a/d Full	AD vector with atg11$^{N544E/I548E/Y551E/L555E/V558E/I562E/Y565E/I569E/T572E}$	This study
pGAD-atg11 a/d-2	AD vector with atg11$^{I548E/Y551E}$	This study
pGAD-atg11 a/d-3	AD vector with atg11$^{L555E/V558E}$	This study
pGAD-atg11 a/d-4	AD vector with atg11$^{I562E/Y565E}$	This study
pGAD-atg11 I562E	AD vector with atg11^{I562E}	This study
pGAD-atg11 Y565E	AD vector with atg11^{Y565E}	This study
pGAD-atg11 I562Q	AD vector with atg11^{I562Q}	This study
pGAD-atg11 I562A	AD vector with atg11^{I562A}	This study
pGAD-atg11 Y565Q	AD vector with atg11^{Y565Q}	This study
pGAD-atg11 Y565A	AD vector with atg11^{Y565A}	This study
pGAD-atg11 I569E	AD vector with atg11^{I569E}	This study
pGBDU-C3	Empty Y2H BD vector	(James et al., 1996)
pGBDU-ATG9	BD vector with full length ATG9	(He et al., 2008)
pGBDU-atg1Δ1-325	BD vector with atg1$\Delta^{1\text{-}325}$ (deletion of the N-terminal kinase domain)	(Abeliovich et al., 2003)

pGBDU-atg11ΔCC2	BD vector with atg11$^{\Delta 536\text{-}576}$	This study
pGBDU-atg11 e/g Full	BD vector with atg11$^{E545A/S547A/R552A/D554A/S559A/A561A/M566A/Q568A/D573A/S575A}$	This study
pGBDU-atg11 a/d-2	BD vector with atg11$^{I548E/Y551E}$	This study
pGBDU-atg11 I562E	BD vector with atg11^{I562E}	This study
pGBDU-atg11 Y565E	BD vector with atg11^{Y565E}	This study
pGBDU-atg11 I562A	BD vector with atg11^{I562A}	This study
pGBDU-atg11 Y565A	BD vector with atg11^{Y565A}	This study
pGBDU-atg11 Y565A	BD vector with atg11^{I569E}	This study
pRS416	Empty pRS416 vector	(Sikorski and Hieter, 1989)
pRS416-Cu-GFP-ATG11	GFP-ATG11 WT driven by the *CUP1* promoter	(Kim et al., 2001)
pRS416-Cu-GFP-Atg11ΔCC2	GFP-Atg11$\Delta^{536\text{-}576}$ driven by the *CUP1* promoter	This Study
pRS416-Cu-GFP-Atg11 e/g Full	GFP-atg11$^{E545A/S547A/R552A/D554A/S559A/A561A/M566A/Q568A/D573A/S575A}$ driven by the *CUP1* promoter	This study
pRS416-Cu-GFP-Atg11 a/d Full	GFP-atg11$^{N544E/I548E/Y551E/L555E/V558E/I562E/Y565E/I569E/T572E}$ driven by the *CUP1* promoter	This study
pRS416-Cu-GFP-Atg11 a/d-4	GFP- atg11$^{I562E/Y565E}$ driven by the *CUP1* promoter	This study
pRS416-Cu-GFP-ATG11 I562E	GFP-Atg11^{I562E} driven by the *CUP1* promoter	This study
pRS416-Cu-GFP-ATG11 Y565E	GFP-Atg11Y^{565E} driven by the *CUP1* promoter	This study
pRS416-Cu-GFP-ATG11 I569E	GFP-Atg11^{I569E} driven by the *CUP1* promoter	This study
pMCSG10	*E. coli* expression vector with T7 promoter and GST tag	(Eschenfeldt et al., 2009)
pMCSG10-ATG11CC2-3	Expression vector with ATG11$^{537\text{-}853}$	This study
pRS414-Cu-PA-ATG19	Protein A tagged ATG19 driven by the *CUP1* promoter	(Yorimitsu and Klionsky, 2005)
pRS414-Cu-ATG9-PA	ATG9 tagged with Protein A, driven by the *CUP1* promoter	This study
pRS414-Cu-HA-ATG11	HA-ATG11 WT driven by the *CUP1* promoter	(Yorimitsu and Klionsky, 2005)

Supplementary Table 2: Strains used in this study

Strain	Genotype	Source
SEY6210	MATα *his3Δ200 leu2-3,112 lys2-801 suc2-Δ9 trp1Δ901 ura3-52*	(Robinson et al., 1988)
atg11Δ	SEY6210 *atg11Δ::KanMX*	This study
ATG1-PA	SEY6210 *atg11Δ::KanMX ATG1-PA::trp*	This study
PJ69-4A	MATα *his3-Δ200 leu2-3,112 trp1-Δ901 ura3-52 gal4Δ gal80Δ LYS2::GAL1-HIS3 GAL2-ADE2 met2::GAL7-lacZ*	(James et al., 1996)
YCY149	MATα *his3-Δ200 leu2-3,112 lys2-801 trp1-Δ901 suc2-Δ9 ura3-52 atg1Δ, 2Δ, 3Δ, 4Δ, 5Δ, 6Δ, 7Δ, 8Δ, 9Δ, 10Δ, 11Δ, 12Δ, 13Δ, 14Δ, 16Δ, 17Δ, 18Δ, 19Δ, 20Δ, 21Δ, 23Δ, 24Δ, 27Δ, 29Δ gal4Δ gal80Δ GAL1-HIS3 GAL2-ADE2 met2::GAL7-lacZ atg31Δ::KanMX*	(Cao et al., 2009)

Supplementary Table 3: Selective media used for Y2H screens

BD partner	AD partner	Selective Media
Atg1$^{\Delta 1\text{-}325}$	Atg11	SMD -ura -leu -ade
Atg9	Atg11	SMD -ura -leu -his with 1mM 3AT
Atg11	Atg11	SMD -ura -leu -ade -his
Atg11	Atg19	SMD -ura -leu -ade

Supplementary Table 4: The BD-Atg11 / AD-Atg11 mutant Y2H screen consistently displayed a background level of AD-11 mutant colonies that still retained the ability to grow on selective plates, even for mutants where most colonies had lost interaction. This background was quantified by replica plating of BD-Atg11 yeast transformed with the indicated AD-Atg11 mutants (two independent plasmid clones per mutant). The number of colonies able to grow on the selective plates was divided by the number of colonies on the non-selective plates to calculate the percentage retained. Although all mutants yielded some colonies that were still able to grow on the selective plates, there was a clear separation between the mutations that caused a loss of interaction and those that did not. The a/d full and a/d-4 mutations allowed only ~20% of colonies to grow on selective plates, while the e/g full and a/d-2 mutations, allowed over 85% to grow on selective plates. Therefore, we concluded that the a/d full and a/d-4 were causing a loss of interaction, consistent with their effects in other screens. The 20% background of apparent revertants was not seen when these Atg11 mutants were screened against any other partner, suggesting that they may be the result of homologous recombination between the mutant AD-Atg11 and the wild type BD-Atg11.

Mutant	Colonies on nonselective plate (-ura -leu)	Colonies retained on selective plate (-ura -leu -his -ade)	Percentage Retained
e/g full clone 1	218	193	88%
e/g full clone 2	235	203	86%
a/d full clone 1	24	4	17%
a/d full clone 2	46	9	20%
a/d-2 clone 1	26	24	92%
a/d-2 clone 2	15	14	93%
a/d-4 clone 1	49	9	18%
a/d-4 clone 2	132	26	20%

Supplementary References

Abeliovich, H., Zhang, C., Dunn, W. A., Shokat, K. M., and Klionsky, D. J. (2003). Chemical Genetic Analysis of Apg1 Reveals A Non-kinase Role in the Induction of Autophagy. *Mol. Biol. Cell* 14, 477–490. doi:10.1091/mbc.e02-07-0413.

Cao, Y., Nair, U., Yasumura-Yorimitsu, K., and Klionsky, D. J. (2009). A multiple ATG gene knockout strain for yeast two-hybrid analysis. *Autophagy* 5, 699–705. doi:10.4161/auto.5.5.8382.

Eschenfeldt, W. H., Lucy, S., Millard, C. S., Joachimiak, A., and Mark, I. D. (2009). A family of LIC vectors for high-throughput cloning and purification of proteins. *Methods Mol. Biol.* 498, 105–115. doi:10.1007/978-1-59745-196-3_7.

He, C., Baba, M., Cao, Y., and Klionsky, D. J. (2008). Self-interaction is critical for Atg9 transport and function at the phagophore assembly site during autophagy. *Mol. Biol. Cell* 19, 5506–16. doi:10.1091/mbc.E08-05-0544.

James, P., Halladay, J., and Craig, E. A. (1996). Genomic Libraries and a Host Strain Designed for Highly Efficient Two-Hybrid Selection in Yeast. *Genetics* 144, 1425–1436. doi:10.1093/GENETICS/144.4.1425.

Kim, J., Kamada, Y., Stromhaug, P. E., Guan, J., Hefner-Gravink, A., Baba, M., et al. (2001). Cvt9/Gsa9 functions in sequestering selective cytosolic cargo destined for the vacuole. *J. Cell Biol.* 153, 381–396. doi:10.1083/jcb.153.2.381.

Robinson, J. S., Klionsky, D. J., Banta, L. M., and Emr, S. D. (1988). Protein sorting in Saccharomyces cerevisiae: isolation of mutants defective in the delivery and processing of multiple vacuolar hydrolases. *Mol. Cell. Biol.* 8, 4936. doi:10.1128/MCB.8.11.4936.

Sikorski, R. S., and Hieter, P. (1989). A system of shuttle vectors and yeast host strains designed for efficient manipulation of DNA in Saccharomyces cerevisiae. *Genetics* 122, 19–27. doi:10.1093/GENETICS/122.1.19.

Yorimitsu, T., and Klionsky, D. J. (2005). Atg11 links cargo to the vesicle-forming machinery in the cytoplasm to vacuole targeting pathway. *Mol. Biol. Cell* 16, 1593–1605. doi:10.1091/mbc.E04-11-1035.
